# Accuracy and Completeness of Intermediate-Level Nursery Descriptions on Hospital Websites

**DOI:** 10.1001/jamanetworkopen.2022.15596

**Published:** 2022-06-06

**Authors:** David C. Goodman, Timothy J. Price, David Braun

**Affiliations:** 1 Dartmouth Institute for Health Policy and Clinical Practice, Geisel School of Medicine at Dartmouth, Lebanon, New Hampshire; 2 Department of Pediatrics, Geisel School of Medicine at Dartmouth, Lebanon, New Hampshire; 3 Department of Community and Family Medicine, Geisel School of Medicine at Dartmouth, Lebanon, New Hampshire; 4 Children’s Hospital at Dartmouth, Lebanon, New Hampshire; 5 Department of Research and Evaluation, Kaiser Permanente Southern California, Pasadena; 6 Department of Pediatrics, Kaiser Permanente Southern California, Panorama City

## Abstract

**Importance:**

Birth at hospitals with an appropriate level of neonatal intensive care units is associated with better neonatal outcomes. The primary sources for information about hospital neonatal unit levels for prospective parents, referring physicians, and the public are hospital websites, but the accuracy of neonatal unit capacity is unclear.

**Objective:**

To determine if hospital websites accurately report the capabilities of intermediate (ie, level II) units, which are intended for care of newborns with low to moderate illness levels or the stabilization of newborns prior to transfer.

**Design, Setting, and Participants:**

This cross-sectional study compared descriptions of level II unit capabilities on hospital web pages in 10 large states with their respective state-level designation. Analyzed units were located in the 10 states with the highest number of live births in 2019 (excluding states with no level II regulations) and had active websites as of May 2021.

**Main Outcomes and Measures:**

Hospital websites were assessed for whether there was any mention of the unit, the description of the unit was provided, the unit was identified as a level III or both levels II and III, the terms “neonatal intensive care unit” or “NICU” were used without indicating limits in care available or newborn acuity, or the unit was claimed to provide the most advanced level of care.

**Results:**

A total 28 states had no regulation of nursery unit levels; in the 10 large, regulated states, web descriptions of level II units were incomplete for 39.2% of hospitals (95% CI, 33.3%-45.3%) and inaccurate for 24.6% (95% CI, 19.6%-30.2%). Within incomplete descriptions, 2.6% (95% CI, 1.1%-5.3%) of hospitals did not mention an advanced care unit and 22.0% (95% CI, 17.2%-27.5%) identified a level II unit without providing further description. Within inaccurate descriptions, 25.4% (95% CI, 20.3%-31.0%) of hospitals described the unit as a “neonatal intensive care unit” or “NICU” without any qualification and 9.3% (95% CI, 6.3%-13.5%) claimed that the unit provided the most advanced neonatal care or care to the sickest newborns; 3.0% of hospitals (95% CI, 1.3%-6.0%) stated that their unit was level III and 1.5% (95% CI, 0.4%-3.8%) as level II and III. Across states there was substantial variation in rates of incompleteness and inaccuracy.

**Conclusions and Relevance:**

Incomplete and inaccurate hospital web descriptions of intermediate newborn care units are common. These deficits can mislead parents, clinicians, and the public about the appropriateness of a hospital for sick newborns, which raises important ethical questions.

## Key Points

**Question:**

How completely and accurately do hospital websites describe their level II special care (ie, intermediate care) nurseries?

**Findings:**

In this cross-sectional study of hospital nurseries (including 1.99 million live births and 268 level II units) in 10 large US states that regulate nursery levels of care, state-designated intermediate (ie, level II) units were inaccurately or incompletely described in 39% and 25% of the hospital websites, respectively. There was substantial and statistically significant variation in rates of incompleteness and inaccuracy across states.

**Meaning:**

These results suggest that hospital websites, often the only source of publicly available information describing a hospital’s neonatal unit, do not provide reliable information for prospective parents, referring physicians, and the public to assess the capacity to care for ill newborns.

## Teaser

This cross-sectional study of hospital neonatal care units in 10 large US states compares state designations of intermediate-care units with how they are described publicly on hospital websites.

## Introduction

Effective regionalization of perinatal care requires that birth and referral hospital capabilities are matched to the risk and illness levels of their patients. For ill newborns, this requires specialized physicians (ie, neonatologists), nurses, and necessary equipment organized in a setting of special care, such as in neonatal intensive care units (NICUs). For over 40 years, the organization and designation of these neonatal units has been guided by a series of policy statements developed through the joint efforts of the March of Dimes and the American Academy of Pediatrics (AAP), with regulations promulgated by many states.^1,2,3,4^ The most recent AAP statement^3^ from 2012 defines 4 levels of care: well newborn nursery (level I), special care nursery (level II), NICU (level III), and regional NICU (level IV).

One aspect of regionalization that has been ignored is the quality of information about NICUs available to parents, referring obstetricians, neonatologists, and to the public at large. While the appropriate site for delivery and newborn care depends on the risk profile of the mother, fetus, or newborn, accurate hospital information is necessary to inform the decision-making process. Many birth hospital websites prominently feature special care or NICUs, presumably to inform families and referring physicians. In most states, the hospital website is the only public information available about a hospital nursery level and its associated capabilities.

The descriptions of intermediate level units (ie, level II) are particularly important. These advanced care nurseries have essential roles in regional systems by providing accessible care for mild and some moderately ill newborns and, when critically ill newborns are born unexpectedly, to stabilize the infant for transport to a higher-level unit. Although the capabilities of these units are appropriate for some ill newborns,^5^ the birth of higher-risk newborns (eg, weighing below 1500 g) have higher average mortality and morbidity than in hospitals with neonatal intensive care units (level III or IV).^6,7,8^ For these reasons, information about hospital nursery capabilities for pregnant patients and referring obstetricians and neonatologists could aid clinical decisions and support national efforts to improve the appropriate location for childbirth. While maternity hospital selection with regards to newborn care capabilities remains very poorly studied, good information is foundational to good decisions. In this study, we report the accuracy and completeness of website descriptions of intermediate care nurseries in relation to a benchmark definition—their designations in their respective states.

## Methods

### State and Hospital Selection

We selected the 10 states with the highest number of live births in 2019, excluding the 28 states that have no level II regulations.^9^ Georgia was one of the top 10 states, but we were unable to obtain a list of hospitals with a designated NICU or special care nursery from the state, and so we substituted in the next ranking state, Virginia. Together, these states (California, Texas, New York, Florida, Illinois, Ohio, Pennsylvania, North Carolina, New Jersey, and Virgina) had 1 990 177 births in 2019, representing 53% of all US births and 66% of births in states with level II regulation (Table 1).

**Table 1.** Level II Advanced Care Nurseries in 10 States With High Numbers of Live Births With State Designation Regulations

State	2019 live births, No.	Total advanced care nurseries	Level II units, No. (%)	Term for unit	Source of level II list
California	446 479	125	14 (11)	Neonatal intensive care unit (intermediate)	Easily available online
Texas	377 599	140	54 (39)	Special care nursery	Easily available online
New York	221 539	76	25 (33)	Level II perinatal center	Easily available online
Florida	220 002	75	35 (47)	Level II neonatal intensive care unit	Required submission of a formal request
Illinois	140 128	96	50 (52)	Perinatal level II or II+	Difficult to find online
Ohio	134 461	54	27 (50)	Level II neonatal care service	Not available online, but provided with call or email
Pennsylvania	134 230	61	21 (34)	Specialty-level facility (level II) or special care nursery	Required submission of a formal request
North Carolina	118 725	55	11 (20)	Level II neonatal service	Not available online, but provided with call or email
New Jersey	99 585	44	23 (53)	Intermediate-care nursery	Difficult to find online
Virginia	97 429	42	8 (19)	Intermediate-level newborn service	Required submission of a formal request
Total	1 990 177	717	268 (37)	NA	NA

Abbreviation: NA, not applicable.

This study relied on publicly available data and did not include human participants, and therefore was not considered to be human participant research as defined by the Dartmouth College institutional review board. The Strengthening the Reporting of Observational Studies in Epidemiology (STROBE) reporting guideline for observational research were followed in this study.

### State Information on Nursery Unit Levels

State lists of hospital nursery levels and regulations were obtained from state government websites and through requests to state agencies. While state terminology used for nursery levels differed, 8 states had a 4-tier system of NICU classification, and these were mapped to AAP levels I through IV. Florida had a 3-tier classification system, with level III encompassing the capabilities of level III and level IV of the AAP. Illinois had 4 tiers with level I, level II, level II+, and level III, which includes the capabilities of AAP levels III and IV. This study examined the reporting of level II and II+ units. The state NICU level terminology was abstracted along with state regulations with regard to level II unit gestational age or weight limits, any additional limits on newborn acuity, limits on care provided, and clinician requirements. Clinician requirements were identified by type of formal training—advanced practice neonatal nurse, pediatrician, or neonatologist. Finally, we noted the steps necessary for the study to obtain each state list of nursery units by level. Units of levels II through IV are referred to as *advanced care units*.

### Hospital Level II Unit Information

For the 268 level II units, we conducted a web search strategy to identify relevant hospital webpages using the terms *maternity*, *obstetrics*, *OB/GYN*, *birthing center*, *women’s health*, *neonatology*, *neonatal intensive care*, *NICU*, *level II*, *special care*, *nursery*, *neonatal*, *high risk*, and *extra care*. For hospitals with website unit descriptions, screen shots were taken with notation of the date and time.^10^ Descriptions were copied verbatim into spreadsheets and were assigned mutually exclusive categories of inaccurate, incomplete, or acceptable independently by 2 study authors (T.J.P. and D.C.G.); differences were discussed and resolved.

Hospital websites were classified as incomplete for 2 reasons: (1) no mention of the unit, or (2) the unit was identified but no description was provided. Websites were classified as inaccurate for 4 reasons: (1) misidentification of a unit as a level III, (2) misidentification of a unit as a level III and level II, (3) misuse of the term *neonatal intensive care unit* or *NICU* without indicating limits in the care available or the degree of prematurity, acuity, and/or complexity of newborns, or (4) description indicating that a unit provides the highest or most advanced level of care without qualifiers (eg, “we provide the highest level of specialized care for premature and critically ill infants”).

### Statistical Analysis

All of the information collected is in the public domain, and the specific hospital descriptions are available in the supplemental materials (eTable 1 in the Supplement). Confidence intervals were calculated using the Clopper-Pearson exact method. Differences of proportions were tested with Fisher exact tests. Analyses were implemented in Stata version 17.0 (Stata Corp). *P* < .05 was considered significant in 2-sided tests.

## Results

Overall, level II units represented 268 of 717 advanced care nurseries (37%), with the proportion ranging from 11% in California (14 of 125 nurseries) to 53% in New Jersey (23 of 44 nurseries) (Table 1). In just 2 states, regulations used the word “intensive” in their definition of level II units. In 3 states, the lists of level II units were easy to find online (California, Texas, New York); in all other states, the information was difficult to find online or was available only with a request to a state agency (ie, each hospital had to be searched in a website or required the direction of a state official to locate). In 3 states (Florida, Pennsylvania, Virginia), a formal open government request was required. The regulations of all states specified either specific gestational age, weight, or illness acuity limits. In all but 2 states, the limits in the duration or type of care were specified. In only 5 states was staffing or immediate availability of a neontologist required (eTable 2 in the Supplement).

Inaccurate or incomplete descriptions were found in two-thirds of hospital website descriptions (68.3%; 95% CI, 57.7%-70.0%), with inaccurate descriptions in 39.2% of websites (95% CI, 33.3%-45.3%) and incomplete descriptions in 24.6% (95% CI, 19.6, 30.2) (Table 2). The most common inaccuracy was the use of the terms “neonatal intensive care” or “NICU” without any qualifier regarding the severity of illness or the care available, which occurred in 25.4% (95% CI, 20.3%-31.0%) of websites. In 9.3% (95% CI, 6.3%-13.5%), the description included language indicating that the level II units provided the most advanced level of care without any qualifier. In 8 instances, or 3.0% (95% CI, 1.3%-6.0%), the website indicated that the unit was a level III unit, and in a further 4 websites the unit was described as being a level II and a level III. The most common incomplete description was identifying a level II unit but without further description, which occurred for 22.0% (95% CI, 17.2, 27.5); 2.6% (95% CI, 1.1%-5.3%) did not mention an advanced care nursery.

**Table 2.** Completeness and Accuracy of Web Descriptions of Level II Intermediate-Care Nurseries for 10 Large States, 2021^a^

Characteristic	No. of units (N = 268)	Total % (95% CI)	Range across 10 large states, %
Inaccurate or incomplete description	171	63.8 (57.7-70.0)	45.5-74.1
Inaccurate description	105	39.2 (33.3-45.3)	10.0-60.0
Identified as level III NICU	8	3.0 (1.3-6.0)	0-11.1
Identified as level II and level III NICU	4	1.5 (0.4-3.8)	0-3.3
Used “neonatal intensive care unit” or “NICU” without indicating limits^b^	68	25.4 (20.3-31.0)	0-48.0
Description indicated that unit provides the highest or most advanced level of care, without qualifiers	25	9.3 (6.3-13.5)	0-17.1
Incomplete description	66	24.6 (19.6-30.2)	8.0-50.0
No mention of any advanced care nursery (ie, level II, III, or IV	7	2.6 (1.1-5.3)	0-10.1
Identified as level II unit but without further description	59	22.0 (17.2-27.5)	8.0-40.0

Abbreviation: NICU, neonatal intensive care unit.

^a^ States examined were California, Texas, New York, Florida, Illinois, Ohio, Pennsylvania, North Carolina, New Jersey, and Virginia with 1 990 177 live births, or 53% of all US births in 2019. Illinois has level II and level II+ categories, and these were analyzed separately. In California, analysis was limited to NICUs participating in the California Children’s Services program, which are the only NICUs that are state regulated. Within these states, level II units were 35% of all advance care nurseries (levels II, III, or IV). Hospital website NICU level information was compared with state NICU level designations (levels I, II, III, or IV).

^b^ Limitations in NICU level included available services or the degree of prematurity, acuity, or complexity of newborns treated.

Across states, there was substantial variation in rates of incompleteness and inaccuracy (Figure). The proportion of hospital websites with inaccurate or incomplete descriptions varied across states, from 45.5% (95% CI, 16.7%-76.6%) in North Carolina to 74.3% (95% CI, 56.7%-87.5%) in Florida, and were unrelated to states’ requirement for in-person accreditation visits (*P* = .09) (eTable 3 in the Supplement). Florida had the highest proportion of inaccurate descriptions (60.0%; 95% CI, 42.1%-76.1%), followed by Texas (55.3%; 95% CI, 41.4%-69.1%) and New York (52.0%; 95% CI, 31.3%-77.2%). Illinois (level II+ units) had the highest proportion of incomplete descriptions (50.0%; 95% CI, 27.2%-72.8%), followed by Pennsylvania (38.1%; 95% CI, 18.1%-61.6%) and Virginia (37.5%; 95% CI, 8.5%-75.5%). Illinois (level II+ units) (10.0%; 95% CI, 1.2%-31.7%) and New Jersey (13.0%; 95% CI, 2.8%-33.6%) had the lowest proportion of inaccurate descriptions. Incomplete descriptions were relatively low in New York (8.0%; 95% CI, 0.01%-26.0%), California (14.3%; 95% CI, 1.8%-42.8%), and Florida (14.3%; 95% CI, 1.8%-42.8%).

**Figure.**
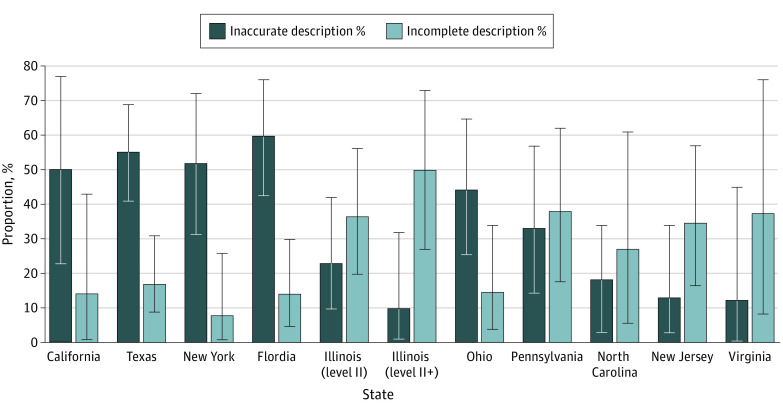
Level II Advanced Care Nurseries With Inaccurate or Incomplete Web Descriptions in 10 Large States, 2021. Differences of proportions across states were tested with Fisher exact tests for incomplete (*P* = .01) and inaccurate (*P* < .001) descriptions.

## Discussion

Realizing the benefits of perinatal regionalization across the US depends upon complex actions taken by federal and state governments and hospitals. The accuracy of hospital-reported newborn care levels is a specific and measurable example of the successes and limitations of regionalization efforts. Kroelinger et al^9,11^ described the heterogenous regulatory status of levels of care, ranging from an absence of any regulation to detailed final rules that define newborn characteristics, unit capability, and breadth of care appropriate for each level. In some states the designation process is the responsibility of each hospital, while in others there is a formal process that requires hospital site visits. Overall, these regulations fall short of current American Academy of Pediatrics Guidelines.^3^ Despite the specificity of these professional guidelines and the regulations in some states, there is no guidance regarding the public description of nursery levels of care.

This study showed that in 10 large states that regulate nursery level of care, almost two-thirds of hospitals provide either inaccurate or incomplete information about their level II units. Categorizing the type and importance of observed deficiencies was difficult. We have primarily used the perspective of what parents would want to know to ensure the health and safety of their newborn. We believe that reasonable parents would expect, and should expect, complete, accurate, and understandable information on whether the needs of their newborn can be met by a hospital. The perinatal care community may also need to more fully educate parents about the differing capabilities of advanced care nurseries. These websites may also be a source of information for perinatal clinicians who are deciding on specific patient referrals or planning referral networks. A level II hospital reporting that they have a higher level of unit than designated by the state or asserting the capability of caring for the very sickest newborn might be judged as egregious in that it gives false reassurance about the capability of care. Not listing a level II unit at all on the hospital website or simply listing a level II unit (or using a similar term) without an accompanying description is not directly misleading but denies relevant information about an important hospital type in systems of regionalization that would be difficult to obtain and interpret from any other source.

One of the intended advantages of level designation stated in the 2004 AAP policy statements on level of neonatal care was that “standardized nomenclature will be informative to the public, especially high-risk maternity patients who seek an active role in selecting a delivery service.”^4^ There are, however, virtually no published studies on the process or information used by parents and clinicians in selecting perinatal hospitals. As such, discussion about the perinatal decision-making process regarding site of care is speculative, reflecting the need for further research. While the process is likely to be different for high- and low-risk patients, complications that change newborn care needs can occur quickly. The possibility that any newborn might need advanced care is familiar to clinicians and, while unstudied in prospective parents, would seem to be generally understood. Previous studies investigating health communication with parents are primarily limited to newborns already admitted to level III or IV units, with the exception of a study of prenatal counselling of midwives in Scotland.^12^ We have found no study that examined the content of information transmitted by hospitals or physicians to prospective parents. It may be that their primary source of information is the family’s obstetrical or primary care clinician. If so, the sources and accuracy of clinicians’ information warrants further research. In the meantime, it appears that hospitals consider their maternity websites important sources of information to the public given their prominence and well-crafted design.

The lack of reporting on NICUs, both in unit level and in quality and outcome measures, is in contrast to other patient populations where public reporting has steadily advanced in scope and quality in the past 30 years, driven by ethical and utilitarian imperatives.^13^ Current sources of health system performance information relevant to other patient populations include Hospital Compare (ie, US Centers for Medicare and Medicaid, the Cystic Fibrosis Foundation, and the Society of Thoracic Surgeons).^14,15,16^ The Leapfrog Group offers comprehensive information about participating hospitals; perinatal measures are limited to jaundice screening of all newborns and a high-risk obstetric measure for newborns with very low birth weight that combines maternal antenatal steroids and delivery at a higher volume hospital.^17^ The Joint Commission’s sole neonatal metric for accreditation status is “unexpected complications in term newborns.”^18^

Beyond ethical reasons, there is evidence that public reporting accelerates quality improvement,^19^ although its value to patients choosing care is dependent on presentation clarity and the efforts made to heighten awareness of its availability.^13,20^ Given the limited information available about NICUs, its value for parents is not known.

The growth of NICU networks (eg, Vermont-Oxford Network and California Perinatal Quality Care Collaborative^21,22^) means that processes of care and outcomes are continuously measured in most US NICUs for high acuity newborns. The data collection for less ill newborns has increased, but less than half of overall NICU admissions are likely to be included in these registries, including a much smaller proportion of newborns cared for in level II units.^23^ Regionalization programs, neonatal network registries, and hospitals have not addressed the pragmatic and ethical aspects of clinical care transparency in strengthening parental and public agency in newborn care.

To the extent that public reporting is discussed within the perinatal community, barriers dominate the dialogue.^24^ The challenges are real and yet differ little from those encountered in other public reporting initiatives with successful implementation. These difficulties include questions about the validity of metrics, the adequacy of risk adjustment, the availability of clinical data gathered by member-based registries, the unintended consequences of less than perfect data, and possible misunderstanding by families. The status quo has its own drawback if it does not challenge misconceptions that every advanced care unit is able handle all types of newborns and provide the highest quality care with the best outcomes.

### Limitations

This study has several limitations. We restricted our study to level II units because of the designation’s unique role in state regulations and the likely importance of this information to care. Claiming that a level I unit had level II or higher capabilities would be a serious inaccuracy, but many states do not regulate these level I units. Inaccurate reporting of a level III or IV unit as a lower-level unit might dissuade parents from selecting the hospital, but the hospitals would have the capacity to provide definitive care to almost all newborns. We did not examine the listing of level III units as level IV because some states do not distinguish between these 2 levels. We also limited the sample to 10 of the 22 states that regulate NICU levels (60% of live births). The nonstudied states have a smaller number of level II units and births. A more important problem is that 28 states have no regulation; evaluating the accuracy of these hospital websites is not feasible. We also assumed that each state’s criteria for level designation was grounded in evidence, but this assumption is challenged by differences in the criteria (eTable 2 in the Supplement). Newborn levels of care are only one part of the perinatal dyad; level of care for pregnant patients is of similar importance and may differ from the hospital neonatal level.^25^ Finally, the study did not attempt to assess the reasons for website deficiencies or whether they are transient or persistent. At the very least, we can report that the hospital websites were viewed twice during a month and did not change substantially. There could be various causes of misreporting, including miscommunication between the clinical unit and hospital marketing staff or an intentional effort to put on the best public face on newborn services. However, it is not known if assessing the reasons for the deficiencies would contribute to improving web content, which would require modest effort by hospitals.

## Conclusions

The concept of perinatal regionalization as a means to better outcomes depends on complex federal, state, and hospital responsibilities that have led to large differences in delivery of care across states, hospitals, and populations. The lack of accurate and family-centered reporting of information that is already available impedes policy development and clinical improvement and denies families and the public of the opportunity to assess hospitals’ neonatal care performance.

## Notes

Supplement. **eTable 1.** Description of Level II Unit on Hospital Website and Assessment Justifications **eTable 2.** Level II Advanced Care Nurseries in the 10 States With Highest Live Births and State Designation Regulations **eTable 3.** Inaccurate and Incomplete Web Descriptions of Level II Advanced Care Nurseries for 10 Large States, 2021
