# Supplementary material for: Accuracy and Completeness of Intermediate-Level Nursery Descriptions on Hospital Websites
Source: JAMA Netw Open. 2022 Jun 6;5(6):e2215596. doi: 10.1001/jamanetworkopen.2022.15596 (PMC9171562; doi:10.1001/jamanetworkopen.2022.15596)
Supplement: Supplement. — eTable 1. Description of Level II Unit on Hospital Website and Assessment Justifications eTable 2. Level II Advanced Care Nurseries in the 10 States With Highest Live Births and State Designation Regulations eTable 3. Inaccurate and Incomplete Web Descriptions of Level II Advanced Care Nurseries for 10 Large States, 2021 [file jamanetwopen-e2215596-s001.pdf]

Supplemental Online Content

Goodman DC, Price TJ, Braun D. Accuracy and completeness of intermediate-level nursery descriptions on hospital websites. *JAMA Netw Open.* 2022;5(6):e2215596. doi:10.1001/jamanetworkopen.2022.15596

eTable 1. Description of Level II Unit on Hospital Website and Assessment Justifications

eTable 2. Level II Advanced Care Nurseries in the 10 States With Highest Live Births and State Designation Regulations

eTable 3. Inaccurate and Incomplete Web Descriptions of Level II Advanced Care Nurseries for 10 Large States, 2021

This supplemental material has been provided by the authors to give readers additional information about their work.

© 2022 Goodman DC et al. *JAMA Network Open.*

eTable 1. Description of Level II Unit on Hospital Website and Assessment Justifications

Hospital Name	City, State	Relevant Descriptors from Hospital Web Sites	Assessment	Last Accessed	Relevant webpage
Clovis Community Medical Center	Clovis CA	"Our Level 2 NICU is designed to meet the needs of premature and/or ill newborns."	INACCURATE Uses the term NICU or Neonatal Intensive Care Unit without indicating limits in the degree of prematurity/acuity/complexity of newborns	5/24/21	Link to webpage
Community Hospital of San Bernardino	San Bernardino CA	"Neonatal Intensive Care Unit (NICU): The Neonatal Intensive Care Unit at Community Hospital of San Bernardino"	INACCURATE – 4	5/24/21	Link to webpage
LPCH Special Care Nursery at Sequoia Hospital	Redwood City CA	"If your baby is premature or requires intensive care"<br><br>"Our Level II Neonatal Intensive Care Unit (NICU) … This NICU"	INACCURATE – 4	5/24/21	Link to webpage
Marin Health Medical Center	Greenbrae CA	"MarinHealth Medical Center level II neonatal intensive care unit (NICU)"	INACCURATE – 4	5/24/21	Link to webpage
Memorial Medical Center	Modesto CA	"Babies born prematurely or with serious health conditions."<br><br>"Specialized care for … premature babies or any infants requiring advanced neonatal care"	INACCURATE Description Indicating that unit provides the highest or most advanced level of care, without qualifiers	5/24/21	Link to webpage
Methodist Hospital of Sacramento	Sacramento CA	"Care for infants born premature or seriously ill … ready to care of the tiniest of newborns."	INACCURATE – 5	5/24/21	Link to webpage
Pioneers Memorial Health Center	Brawley, CA	Pioneers Memorial Hospital has both an adult ICU and a level 2 neonatal care unit"	INCOMPLETE– 2	5/24/21	Link to webpage
Rady Children's at Scripps Memorial	San Diego, CA	"Level II neonatal units provide support and care for babies who have mild to moderate health problems at birth."	ACCURATE	5/24/21	Link to Webpage

© 2022 Goodman DC et al. *JAMA Network Open.*

Hospital Encinitas					
Rady Children's at Scripps Mercy Hospital Chula Vista	Chula Vista, CA	Same description as above.	ACCURATE	5/24/21	Link to webpage
Rady Children's at Scripps Mercy Hospital San Diego	San Diego, CA	Same description as above.	ACCURATE	5/24/21	Link to webpage
Sharp Chula Vista Medical Center	Chula Vista, CA	"Superior neonatal intensive care for our tiniest patients … your baby is in the best possible hands."	INACCURATE – 5	5/24/21	Link to webpage
UC San Diego Medical Center - Hillcrest	Hillcrest, CA	"Level II Intermediate NICU for babies who need short-term intensive care after birth""	ACCURATE	5/24/21	Link to webpage
Washington Hospital	Fremont CA	"...specialize in caring for infants who are born prematurely at 32 weeks' gestation or greater or are underweight or are moderately ill. Additionally, babies who are born more premature than 32 weeks' gestation are transferred back to a level II nursery for convalescent care after being treated in intensive care units."	ACCURATE	5/24/21	Link to webpage
Watsonville Community Hospital	Watsonville, CA	"We offer: Intermediate intensive care nursery"	INCOMPLETE – 2	5/24/21	Link to webpage
Ascension Providence	Waco, TX	"Ascension Providence delivers advanced care in our neonatal intensive care unit (NICU)"	INACCURATE – 4	5/24/21	Link to webpage
Ascension Seton Hays	Kyle, TX	"…work together to deliver care for babies who are born as early as 32 weeks"	ACCURATE	5/24/21	Link to webpage
Ascension Seton Northwest	Austin, TX	"Our neonatology nurses and neonatologists deliver care for babies who are born as early as 32 weeks"	ACCURATE	5/24/21	Link to webpage
Baptist Hospitals of Southeast Texas	Beaumont, TX	"Our NICU is committed to helping your new baby get emergency care immediately."	INACCURATE – 4	5/24/21	Link to webpage
Baptist Medical Center	San Antonio, TX	"Level IIIA NICU: Baptist Medical Center"	INACCURATE – 1	5/24/21	Link to webpage

© 2022 Goodman DC et al. *JAMA Network Open.*

Baylor Scott & White Medical Center - Centennial	Frisco, TX	“Centennial is home to a Level II neonatal intensive care unit (NICU) to provide advanced care to babies”	INACCURATE – 4	5/24/21	Link to webpage
Baylor Scott & White Medical Center - Frisco	Frisco, TX	“Prepared to provide advanced life-support services and technologies for premature and seriously ill newborns … Our Level II NICU provides the highest level of care available for our tiniest patients”	INACCURATE – 5	5/24/21	Link to webpage
Baylor Scott & White Medical Center – Irving	Irving, TX	“provides advanced life-support services and technologies for premature and seriously ill newborns … specialized training to care for premature and critically ill newborns”	INACCURATE – 5	5/24/21	Link to webpage
Baylor Scott & White Medical Center – Lake Pointe	Rowlett, TX	“provides advanced life-support services and technologies for premature and seriously ill newborns … specialized training to care for premature and critically ill newborns”	INACCURATE – 5	5/24/21	Link to webpage
Cedar Park Regional Medical Center	Cedar Park, TX	“Cedar Park Regional Medical Center offers a Level II Neonatal Intensive Care Unit”	INACCURATE – 4	5/24/21	Link to webpage
CHRISTUS Mother Frances Hospital - Sulphur Springs	Sulpher Springs, TX	No reference to unit.	INCOMPLETE – 1	4/22/21	Link to Hospital Web site
CHRISTUS Santa Rosa - New Braunfels	New Braunfels, TX	“Newborns with medically complicated diagnoses have direct access to the highly trained Emergency Transport Team so they can receive the highest level NICU care at The Children's Hospital of San Antonio”	ACCURATE	5/24/21	Link to webpage
CHRISTUS Santa Rosa Hospital - Westover Hills	San Antonio, TX	“for a wide range of babies who need additional intervention, but typically don’t have life threatening conditions”	ACCURATE	5/24/21	Link to webpage
Citizens Medical Center	Victoria, TX	“with a well-baby nursery as well as a Level II neonatology unit”<br><br>“home to a Level III (B) neonatology unit”	INACCURATE – 2	5/24/21	Link to webpage
City Hospital at White Rock	Dallas, TX	No reference to unit.	INCOMPLETE – 1	4/22/21	Link to Hospital Web site

© 2022 Goodman DC et al. *JAMA Network Open.*

DeTar Healthcare System	Victoria, TX	“DeTar Healthcare System's 9-bed Level II neonatal intensive care unit (NICU)”	INACCURATE – 4	5/24/21	Link to webpage
Harlingen Medical Center	Harlingen, TX	"Designated Level II Nursery"	INCOMPLETE – 2	5/24/21	Link to webpage
HCA Houston Healthcare Conroe	Conroe, TX	“22 bed NICU provides intermediate care for 32 weeks GA or greater”	ACCURATE	5/24/21	Link to webpage
HCA Houston Healthcare Tomball	Tomball, TX	“11 bed NICU provides intermediate care for your sick or pre-term newborn…. For infants born less than 32 weeks”	ACCURATE	5/24/21	Link to webpage
HCA Houston Healthcare West	Houston, TX	“eight-bed Level II NICU cares for infants born less than 32 weeks gestation, have a low birth weight, experienced difficulties during delivery or show signs of a problem shortly after birth"	ACCURATE	5/24/21	Link to webpage
Houston Methodist Baytown Hospital	Baytown, TX	"Level II special care nursery"	INCOMPLETE – 2	5/24/21	Link to webpage
Houston Methodist Clear Lake Hospital	Houston, TX	“We are the only hospital in the Bay Area with a Level II NICU”	INACCURATE – 4	5/24/21	Link to webpage
Houston Methodist Hospital	Houston, TX	“level II special care nursery”	INCOMPLETE – 2	5/24/21	Link to webpage
Houston Methodist Sugar Land Hospital	Sugar Land, TX	“our level II NICU … Babies who require a higher level of care are stabilized and transported”	ACCURATE	5/24/21	Link to webpage
Knapp Medical Center	Weslaco, TX	No reference to unit.	INCOMPLETE – 1	4/22/21	Link to webpage
Medical City Frisco, a Medical Center of Plano Facility	Frisco, TX	“Medical City Frisco’s Level II NICU provides specialty care for stable or moderately ill newborns.”	INCOMPLETE – 2	5/24/21	Link to webpage
Medical City McKinney	McKinney TX	“offering special care for babies born at 26-weeks gestation or later”<br><br>*This violates TX regulations for GA cared for in Level II Units	INACCURATE – 5	5/24/21	Link to webpage

© 2022 Goodman DC et al. *JAMA Network Open.*

Memorial Hermann Cypress Hospital	Cyprus, TX	"A Level II nursery providing 24-hour neonatal coverage"	INCOMPLETE – 2	5/24/21	Link to webpage
Memorial Hermann Greater Heights Hospital	Houston, TX	"Level II nursery… cares for all but the most critically ill newborns"	ACCURATE	5/24/21	Link to webpage
Memorial Hermann Katy Hospital	Katy, TX	"Memorial Hermann Katy Hospital's Level III Neonatal Intensive Care Unit (NICU) is equipped to provide specialized care"	INACCURATE – 1	5/24/21	Link to webpage
Memorial Hermann Northeast	Humble, TX	"our facility includes Level II and Level III Neonatal Intensive Care Units (NICU) for infants as young as 28 weeks gestation"	INACCURATE – 2	5/24/21	Link to webpage
Memorial Hermann Southeast Hospital	Houston, TX	"Our Level III NICU provides the full range of care for neonates"	INACCURATE – 1	5/24/21	Link to webpage
Memorial Hermann Sugar Land Hospital	Sugar Land, TX	"Level II-B Neonatal Intensive Care Unit"	INACCURATE – 4	5/24/21	Link to webpage
Methodist Charlton Medical Center	Dallas, TX	"Specialized infant care services: Level II Nursery"	INCOMPLETE – 2	5/24/21	Link to webpage
Methodist Mansfield Medical Center	Mansfield, TX	"an eight-bed Neonatal Intensive Care Unit (NICU)"	INACCURATE – 4	5/24/21	Link to webpage
Methodist Stone Oak Hospital	San Antonio, TX	"Level II NICU. Your newborn will be under the watch of a dedicated team of registered nurses, neonatologists and a 24-hour on-site nurse practitioner"	INCOMPLETE – 2	5/24/21	Link to webpage
Midland Memorial Hospital	Midland, TX	"Designated as a Level II Neonatal Intensive Care Unit (NICU)"	INACCURATE – 4	5/24/21	Link to webpage
Nacogdoches Medical Center	Nacogdoches, TX	"Our level II NICU has the equipment and medical staff capable of providing care for newborns with the following conditions: …"	ACCURATE	5/24/21	Link to webpage

© 2022 Goodman DC et al. *JAMA Network Open.*

Nacogdoches Memorial Hospital	Nacogdoches, TX	“With our state-of-the-art facility, we offer the highest level of care for babies with a gestational age of at least 30 weeks:”	ACCURATE	5/24/21	Link to webpage
Northeast Baptist Hospital	San Antonio, TX	“Level IIIA NICUs: Northeast Baptist Hospital”	INACCURATE – 1	5/24/21	Link to webpage
OakBend Medical Center	Richmond, TX	“…provides exceptional Neonatal Care for: Infants born at 32 weeks gestation or older, or a weight of 1500g (3.3 lbs) or greater, who are moderately ill”	ACCURATE	5/24/21	Link to webpage
Resolute Health	New Braunfels, TX	“Level II NICU - … our neonatologists and NICU care team are ready and able to provide that care”	INACCURATE – 4	5/24/21	Link to webpage
Round Rock Medical Center	Round Rock, TX	“Level II NICU”	INACCURATE – 4	5/24/21	Link to webpage
Shannon Medical Center	San Angelo, TX	“if your baby should need more specialized care, our Special Care Nursery is equipped … to care for babies with special needs.”	ACCURATE	5/24/21	Link to webpage
St. David's South Austin Medical Center	Austin, TX	“Level II NICU”	INACCURATE – 4	5/24/21	Link to webpage
St. Luke's Hospital at The Vintage	Houston, TX	"...featuring 14 private labor, delivery, recovery and postpartum rooms and a Level II Neonatal Intensive Care Unit (NICU)."	INCOMPLETE – 2	5/24/21	Link to webpage
St. Luke's Sugar Land Hospital	Sugar Land, TX	“Newborns who need extra medical care stay in our Level II Neonatal Intensive Care Unit (NICU).”	INACCURATE – 4	5/24/21	Link to webpage
St. Luke's The Woodlands Hospital	The Woodlands, TX	“…depending on your baby’s needs, there are different levels of care. Newborns who need extra medical care stay in our Level II NICU”	ACCURATE	5/24/21	Link to webpage
Texas Health Harris Methodist Hospital Alliance	Fort Worth, TX	"Level II NICU to care for your baby after your birth"	INACCURATE – 4	5/24/21	Link to webpage
Texas Health Harris Methodist Hospital Hurst-Euless-Bedford	Bedford, TX	"Level II NICU to care for your baby after your birth"	INACCURATE – 4	5/24/21	Link to webpage
Texas Health Harris Methodist Hospital Southwest Fort Worth	Fort Worth, TX	“**Level III NICU to care for your baby after your birth.**”	INACCURATE – 2	5/24/21	Link to webpage

© 2022 Goodman DC et al. *JAMA Network Open.*

Texas Health Presbyterian Hospital Allen	Allen, TX	""a Level II NICU to care for your baby after your birth."	INACCURATE – 4	5/24/21	Link to webpage
Texas Health Presbyterian Hospital Flower Mound	Flower Mound, TX	"Our Level II NICU provides an advanced, high level of care for at-risk babies"	INACCURATE – 4	5/24/21	Link to webpage
Texoma Medical Center	Denison, TX	"Texoma region's first and only Level II NICU"	INACCURATE – 4	5/24/21	Link to webpage
The Medical Center of Southeast Texas	Port Arthur, TX	"Our Level II NICU (neonatal intensive care unit) provides a higher level of care for babies who are sick or who need special medical treatment"	INACCURATE – 4	5/24/21	Link to webpage
Titus Regional Medical Center	Mount Pleasant TX	"Neonatal Intensive Care Unit for infants needing more intensified care and treatment."	INACCURATE – 4	5/24/21	Link to webpage
United Regional Health Care System	Wichita Falls, TX	"As a Level II Special Care Nursery designated facility, United Regional is able to provide care for infants with a 30-week or longer gestational age and a birth weight of greater than 1,250 grams"	ACCURATE	5/24/21	Link to webpage
Wadley Regional Medical Center	Texarkana TX	"Our nursery provides newborn, transition and Level II intensive care with 24-hour neonatology service."	INCOMPLETE – 2	5/24/21	Link to webpage
Wise Health System	Decatur, TX	"for infants with moderate risk of serious complications related to immaturity, illness"	ACCURATE	5/24/21	Link to webpage
Woodland Heights Medical Center	Lufkin, TX	"Our Level II NICU offers specialized services for newborns in need of additional care."	INACCURATE – 4	5/24/21	Link to webpage
St. Charles Hospital	Port Jefferson NY	"…the NICU, provides state-of-art care for high-risk newborns, including babies with a low birth weight and premature babies born as early as 32 weeks."	ACCURATE	5/24/21	Link to webpage
St Luke's Cornwall Hospital/Newburgh	Newburgh, NY	"…the NICU provides medical care to critically ill babies and offers special therapeutic services, community referrals, family education, and discharge planning."	INACCURATE – 4	5/24/21	Link to webpage
St Johns Episcopal Hospital So Shore	Far Rockaway, NY	"The Level II (special care) nursery, located on the same floor, accommodates up to 25 newborns and is staffed by registered nurses who are specially trained in newborn care.""	INCOMPLETE – 2	5/24/21	Link to webpage

© 2022 Goodman DC et al. *JAMA Network Open.*

St Catherine of Siena Hospital	Smithtown NY	"Level II Neonatal Intensive Care Unit: The new seven-bed unit is designed to provide specialized care for your newborn"	INACCURATE – 4	5/24/21	Link to webpage
SJRH - St Johns Division	Yonkers, NY	"Our state-of-the-art, Level II, Neonatal Intensive Care Nursery is equipped to provide comprehensive medical services if your baby is born prematurely or has other special needs.""	INACCURATE – 4	5/24/21	Link to webpage
Samaritan Medical Center	Watertown, NY	"We are the only Level II NICU north of Syracuse … babies delivered at 32 weeks and older that need a little extra care before they can go home."	ACCURATE	5/24/21	Link to webpage
Rochester General Hospital	Rochester, NY	"Should your baby require additional attention or special care, we have a Level II Special Care Nursery on site with neonatologists and pediatricians available."	INCOMPLETE – 2	5/24/21	Link to webpage
NYU Langone Hospital-Brooklyn	Brooklyn, NY	"Level II Perinatal Center … care for infants born at 32 weeks gestation or older, who weigh at least 1,500 grams (roughly 3.3 pounds), and are considered moderate risk"	ACCURATE	5/24/21	Link to webpage
North Central Bronx Hospital	Bronx, NY	"Level II NICU"	INACCURATE – 4	5/24/21	Link to webpage
New York-Presbyterian/Lower Manhattan Hospital	New York, NY	"Newborns who are premature or who require medical or surgical management immediately following birth receive expert care in the Hospital's Neonatal Intensive Care Unit	INACCURATE – 4	5/24/21	Link to webpage
New York-Presbyterian/Hudson Valley Hospital	Cortlandt Manor, NY	"NICU is located on the unit for premature infants (born 30 weeks or later into the pregnancy) and those with specialized needs ... If your baby is born earlier than 30 weeks, we will transfer him or her to another hospital"	ACCURATE	5/24/21	Link to webpage
New York-Presbyterian Lawrence Hospital	Bronxville, NY	"Level 2 Neonatal Intensive Care Unit for premature infants and those with specialized needs"	INACCURATE – 4	5/24/21	Link to webpage
New York-Presbyterian Hospital - Allen Hospital	New York, NY	"we care for premature infants and those with respiratory distress, jaundice, blood sugar problems, and other difficulties in our more advanced NICU Nursery"	INACCURATE – 4	5/24/21	Link to webpage
Mount Sinai South Nassau	Oceanside, NY	"Our Level II NICU experts will join your health care team seamlessly to provide expert care"	INACCURATE – 4	5/24/21	Link to webpage

© 2022 Goodman DC et al. *JAMA Network Open.*

Montefiore Nyack	Nyack, NY	"The Intermediate Care Nursery (ICN) at Montefiore Nyack Hospital provides the highest standard of care to newborns who require specialized care."	INACCURATE – 5	5/24/21	Link to webpage
Millard Fillmore Suburban Hospital	Amherst, NY	"This special care nursery provides care to infants weighing more than 3lbs, 5 oz and/or more than 32 weeks gestation who are moderately ill."	ACCURATE	5/24/21	Link to webpage
Mercy Hospital of Buffalo	Buffalo, NY	"we have a Level II NICU, for babies who are born after 32 weeks gestation or who are recovering from more serious conditions	ACCURATE	5/24/21	Link to webpage
Long Island Jewish Forest Hills	Forest Hills, NY	"Level 2 Neonatal Intensive Care Unit (NICU) Neonatologist on site 24/7"	INACCURATE – 4	5/24/21	Link to webpage
Huntington Hospital	Huntington, NY	"… a Level 2 Neonatal Intensive Care Unit—so you can rest assured when your baby needs extra-special care. "	INACCURATE – 4	5/24/21	Link to webpage
Good Samaritan Hospital of Suffern	Sufferen, NY	"provides the specialized, multidisciplinary special care services for infants as young as 30 weeks and as small as 2 1/2lbs. at birth."	ACCURATE	5/24/21	Link to webpage
Garnet Health Medical Center	Middletown, NY	"10-bed, Level II Neonatal Intensive Care Unit… care for your premature and sick infants requiring specialty care"	INACCURATE – 4	5/24/21	Link to webpage
Faxton-St Lukes Healthcare St Lukes Division	Utica, NY	"The only Special Care Level II Nursery in the Utica area. The nursery is ready with the best equipment, specialized physicians known as neonatologists and an excellent nursing staff."	ACCURATE	5/24/21	Link to webpage
Ellis Hospital - Bellevue Woman's Care Center	Niskayuna, NY	"Bellevue's Level II Special Care Nursery is one of only three in northeastern New York that provides these specialized services for infants over 30 weeks gestation in need of non-surgical medical intervention and care."	ACCURATE	5/24/21	Link to webpage
Coney Island Hospital	Brooklyn, NY	"Level II Neonatal Intensive Care Unit (NICU), which uses some of the latest monitoring and ventilating machines."	INACCURATE – 4	5/24/21	Link to webpage
Cayuga Medical Center at Ithaca	Ithaca, NY	"Our certified Level 2 Neonatal Intensive Care Unit (NICU) … admits babies born up to 10 weeks early (30 weeks and above) and at a minimum weight of 1250 grams (2 pounds, 12 ounces"	ACCURATE	5/24/21	Link to webpage

© 2022 Goodman DC et al. *JAMA Network Open.*

AdventHealth Altamonte Springs	Altamonte Springs, FL	"Expert Care and Compassion For the Tiniest Babies … At AdventHealth Altamonte Springs, we're prepared for all eventualities"	INACCURATE – 5	6/9/21	Link to webpage
AdventHealth Celebration	Celebration, FL	"Expert Care and Compassion For the Tiniest Babies … At AdventHealth Altamonte Springs, we're prepared for all eventualities"	INACCURATE – 5	6/9/21	Link to webpage
AdventHealth Daytona Beach	Daytona Beach, FL	"Expert Care and Compassion For the Tiniest Babies … At AdventHealth Altamonte Springs, we're prepared for all eventualities"	INACCURATE – 5	6/9/21	Link to webpage
AdventHealth Ocala	Ocala, FL	"The NICU is designed to care for babies born at or after 32 weeks who weigh more than 3.3 pounds, as well as babies with certain health problems"	ACCURATE	6/9/21	Link to webpage
AdventHealth Winter Park	Winter Park, FL	"Expert Care and Compassion For the Tiniest Babies … At AdventHealth Altamonte Springs, we're prepared for all eventualities"	INACCURATE – 5	6/9/21	Link to webpage
Ascension Sacred Heart of the Emerald Coast	Miramar Beach, FL	"This 10-bed, Level II unit cares for infants born prematurely, up to two months early, who weigh more than 3.3 pounds"	ACCURATE	6/9/21	Link to webpage
Ascension St. Vincent's Riverside	Jacksonville, FL	"Level 2 Neonatal Intensive Care Unit (NICU) for babies with special needs and medical specialists on staff 24-hours a day to ensure your baby has the highest level of care"	INACCURATE – 5	6/9/21	Link to webpage
Baptist Medical Center South	Jacksonville, FL	"Our expert team at Wolfson Children's also operates a Level 2 NICU at Baptist Medical Center South."	INACCURATE – 4	6/9/21	Link to webpage
Bayfront Health Port Charlotte	Port Charlotte, FL	"Our NICU is able to care for premature babies who weigh 2 lbs. 4 oz. or more."	ACCURATE	6/9/21	Link to webpage
Bayfront Health Spring Hill	Spring Hill, FL	"The 10-bed Level II NICU offers exclusive services and specially trained providers … Services are for babies that need a little extra support and access to clinical resources that may enhance quality of care and increase their safety."	ACCURATE	6/9/21	Link to webpage
Boca Raton Regional Hospital	Boca Raton, FL	No reference to unit.	INCOMPLETE – 1	6/9/21	Link to Hospital Web site
Broward Health Coral Springs	Coral Springs, FL	"Level II Neonatal Unit with private suites and sleep accommodations for a parent to sleep"	INCOMPLETE – 2	6/9/21	Link to webpage

© 2022 Goodman DC et al. *JAMA Network Open.*

Cleveland Clinic Martin North Hospital	Stuart, FL	"Martin North Hospital and Tradition Hospital have a Level II Special Care Nursery should your baby have special needs after birth."	INCOMPLETE – 2	6/9/21	Link to webpage
Cleveland Clinic Tradition Hospital	Point Saint Lucie, FL	Same description as above.	INCOMPLETE – 2	6/9/21	Link to webpage
Flagler Hospital	Saint Augustine, FL	"The focus of our NICU is to meet the needs of the entire family in a supportive environment. The NICU combines intensive care with developmentally based care"	INACCURATE – 4	6/9/21	Link to webpage
Fort Walton Beach Medical Center	Fort Walton Beach, FL	"We are the only Level II Neonatal Intensive Care Unit (NICU) in the Tri-County area accepting newborns as young as 28 weeks, or 1000gms"	ACCURATE	6/9/21	Link to webpage
Good Samaritan Medical Center	West Palm Beach, FL	"an on-site Level II NICU which is staffed with the specialists your baby may need if intensive care is required. The entire NICU health care team works to provide some of the best care for sick or at-risk newborns"	INACCURATE – 4	6/9/21	Link to webpage
Hialeah Hospital	Hialeah FL	This hospital ended their Labor and Delivery services in April 2021.	ACCURATE	6/9/21	Link to webpage
Holmes Regional Medical Center	Melbourne, FL	"Amenities include: Level II NICU located at Holmes Regional Medical Center"	INACCURATE – 4	6/9/21	Link to webpage
Holy Cross Hospital	Fort Lauderdale, FL	No reference to unit.	INCOMPLETE – 1	6/9/21	Link to Hospital web site
Jackson North Medical Center	North Miami Beach, FL	"the Level 2 NICU at Jackson North Medical Center is close to the maternity unit in the Women's Pavilion"	INACCURATE – 4	6/9/21	Link to webpage
Jupiter Medical Center	Jupiter, FL	"Jupiter Medical Center's Level II De George Neonatal Intensive Care Unit (NICU) is equipped to care for babies born as early as 32 weeks"	ACCURATE	6/9/21	Link to webpage
Manatee Memorial Hospital	Bradenton, FL	"The Manatee Memorial Hospital's Neonatal Intensive Care Unit (NICU) is Manatee County's only Level II NICU and has the advanced technology to care for tiny and special care babies."	INACCURATE – 4	6/9/21	Link to webpage
Medical Center of Trinity	Trinity, FL	"NICU: The neonatal intensive care unit provides critical care to newborns who are ill or premature. Many factors may lead to a newborn being	INACCURATE – 4	6/9/21	Link to webpage

© 2022 Goodman DC et al. *JAMA Network Open.*

		admitted to the NICU, including low birth weight or complications during delivery. "			
Memorial Hospital Jacksonville	Jacksonville, FL	No reference to unit.	INCOMPLETE – 1	6/9/21	Link to Hospital web site
Memorial Hospital Miramar	Miramar, FL	Level II NICUs for babies requiring neonatal care … Babies can stay in the NICU anywhere from a few days to more than six months, depending on their care needs.”	INACCURATE – 4	6/9/21	Link to webpage
Memorial Hospital West	Pembroke Pines, FL	Same description as above.	INACCURATE – 4	6/9/21	Link to webpage
Mercy Hospital Plantation	Miami, FL	“Mercy Hospital is home to a fully-accredited, Level III Neonatal Intensive Care Unit”	INACCURATE – 1	6/9/21	Link to webpage
Morton Plant Hospital	Clearwater, FL	“Level II NICU on-site with private rooms"	INACCURATE – 4	6/9/21	Link to webpage
NCH Healthcare System North Naples Hospital	Naples, FL	“18 bed Level II NICU with state-of-the-art technology staffed with 24-hour on-site neonatologists for babies with complications. The NICU within the BirthPlace is the only neonatal intensive care unit in Collier County.”	INACCURATE – 4	6/9/21	Link to webpage
Oak Hill Hospital	Brooksville, FL	“Our neonatal intensive care unit is fully equipped to provide the extra special care and attention necessary for the babies with the most serious needs … NICU team members to provide specialized care for our tiniest patients”	INACCURATE – 5	6/9/21	Link to webpage
Orange Park Medical Center	Orange Park, FL	“Orange Park Medical Center is home to a neonatal intensive care unit (NICU) where newborns in need of extra attention following birth receive additional medical support”	INACCURATE – 4	6/9/21	Link to webpage
Palmetto General Hospital	Hialeah, FL	“For infants with special needs, around-the-clock neonatal care is available in our licensed 15-bassinet, Level II Intensive Care Unit.”	INACCURATE – 4	6/9/21	Link to webpage
Palms West Hospital	Loxahatchee, FL	“Our Level II Neonatal Intensive Care Unit (NICU) offers specialized medical care to critically ill infants, delivered 24 hours a day by board-certified neonatologists and an experienced nursing team”	INACCURATE – 4	6/9/21	Link to webpage

© 2022 Goodman DC et al. *JAMA Network Open.*

Rockledge Regional Medical Center	Rockledge, FL	No reference to unit.	INCOMPLETE – 1	6/9/21	Link to webpage
St. Joseph's Hospital South	Riverview, FL	"A level II neonatal intensive care unit (NICU) is open at St. Joseph's Hospital-South. In the event that your baby needs an even higher-level care, we can transport them to our level IV NICU at St. Joseph's Women's Hospital."	ACCURATE	6/9/21	Link to webpage
Winter Haven Women's Hospital	Winter Haven, FL	"A Level II NICU can provide care for babies born at 28 weeks or later who weigh more than 1000 grams (2.2 pounds); who might have inability to maintain body temperature; who may have difficulty with oral feedings; who are moderately ill"	ACCURATE	6/9/21	Link to webpage
Advocate BroMenn Medical Center (Level II)	Normal, IL	No reference to unit.	INCOMPLETE – 1	5/26/21	Link to Hospital web site
Advocate South Suburban Hospital (Level II)	Hazel Crest, IL	"Just steps away from our birthing suites, Advocate South Suburban houses a Level II intermediate care nursery, which offers superior care for newborns and premature babies."	INCOMPLETE – 2	5/26/21	Link to webpage
Advocate Trinity Hospital (Level II)	Chicago, IL	"Comprehensive testing and monitoring and a Level II+ special care nursery assures that you and your baby will be well cared for."	INACCURATE – 1	5/26/21	Link to webpage
Alton Memorial Hospital (Level II)	Alton, IL	No reference to unit.	INCOMPLETE – 1	5/26/21	Link to Hospital web site
AMITA Health Adventist Medical Center Bolingbrook (Level II)	Bolingbrook, IL	"Level II Perinatal Services with an Intermediate Care Nursery"	INCOMPLETE – 2	5/26/21	Link to webpage
AMITA Health Adventist Medical Center Glen Oaks (Level II)	Glendale Hts, IL	"Level II Perinatal Services, with doctors from Lurie Children's Hospital in Chicago"	INCOMPLETE – 2	5/26/21	Link to webpage
Amita Health Mercy Medical	Aurora, IL	""Level II Perinatal Services, with doctors from Lurie Children's Hospital in Chicago"	INACCURATE – 2	5/26/21	Link to webpage

© 2022 Goodman DC et al. *JAMA Network Open.*

Center Aurora (Level II)		"Our Level II+ Perinatal Services offers the same care capabilities as Level II, but with the added support of a special care nursery.”			To second descriptor
Anderson Hospital (Level II)	Mayville, IL	“Our advanced Level II newborn nursery assures parents that we can care for their new baby even in the unlikely event of a more complicated birth.”	INCOMPLETE – 2	5/26/21	Link to webpage
Blessing Hospital (Level II)	Quincy, IL	"If your baby requires a higher level of care, our level II specialty care nursery provides additional monitoring and special attention. We work closely with providers at hospitals with neonatal intensive care units to quickly transfer babies who require a more advanced level of care."	ACCURATE	5/26/21	Link to webpage
CGH Medical Center (Level II)	Sterling, IL	No reference to unit.	INCOMPLETE – 1	5/26/21	Link to webpage
Decatur Memorial Hospital (Level II)	Decatur, IL	“Having a Level II Special Care Nursery means we are equipped to manage complications that regular nurseries cannot … If an infant needs continuous ventilation, they will need to be transferred to a Level III NICU”	ACCURATE	5/26/21	Link to webpage
FHN Memorial Hospita (Level II)l	Freeport, IL	No reference to unit.	INCOMPLETE – 1	5/26/21	Link to webpage
Gateway Regional Medical Center (Level II)	Granite City, IL	No reference to unit.	INCOMPLETE – 1	5/26/21	Link to webpage.
Genesis Medical Center-Silvis (Level II)	Silvis, IL	“The Silvis BirthCenter has a Level II Special Care Nursery. Your child also has access to services that include: …. Neonatal transport services”	ACCURATE	5/26/21	Link to webpage
Graham Hospital (Level II)	Canton, IL	"We proudly support a Level II nursery, meaning at Graham Hospital we are equipped and capable of caring for infants 32 weeks gestation and greater. Should you go into labor or deliver before 32 week … transfer to a Level III facility can occur."	ACCURATE	5/26/21	Link to webpage
HSHS St Anthony's Memorial	Effingham, IL	No reference to unit.	INCOMPLETE – 1	5/26/21	Link to webpage

© 2022 Goodman DC et al. *JAMA Network Open.*

Hospital (Level II)					
HSHS St Elizabeth's Hospital (Level II)	O'Fallon, IL	"The Intermediate Care Nursery provides private rooms with a recliner, sleeper sofa, and refrigerator for breastmilk storage, which allows family to bond with their baby and participate in his or her care"	INCOMPLETE – 2	5/26/21	Link to webpage
HSHS St Joseph's Hospital (Level II)	Breese, IL	Level II nursery for: Moderate-risk newborns who don't require intensive care; Infants with low birth weight or mild to moderate respiratory distress at birth; Babies transferred from Level III (Tertiary Care) facilities. Should the need arise, Level II nursery staff are trained to stabilize infants who need to be transferred to a tertiary care hospital."	ACCURATE	5/26/21	Link to webpage
HSHS St Mary's Hospital (Level II)	Decatur, IL	"in our special care nursery, we can: stabilize high-risk and/or premature infants; Transport babies to a Level III Neonatal Intensive Care Unit (NICU) at our sister hospital St. John's Children's Hospital in Springfield"	ACCURATE	5/26/21	Link to webpage
Illinois Valley Community Hospital (Level II)	Peru, IL	No reference to unit.	INCOMPLETE – 1	5/26/21	Link to Hospital web site
Katherine Shaw Bethea Hospital (Level II)	Dixon, IL	No reference to unit.	INCOMPLETE – 1	5/26/21	Link to Hospital Web site
MacNeal Hospital (Level II)	Berwyn, IL	"Level II neonatal nursery – capable of caring for late-preterm infants, from gestation 32 weeks to 37 weeks"	ACCURATE	5/26/21	Link to webpage
McDonough District Hospital (Level II)	MaComb, IL	No reference to unit.	INCOMPLETE – 1	5/26/21	Link to Hospital Web site
Memorial Medical Center (Level II)	Springfield, IL	"The Level II Nursery offers advance newborn care for babies born at greater than 32 weeks gestation who may need 24-hour monitoring or a higher level of nursing care."	ACCURATE	5/26/21	Link to webpage
Morris Hospital & Healthcare Centers (Level II)	Morris, IL	"As a Level II Perinatal Care Provider, we have the necessary qualifications to care for women who have the potential for complicated or high-risk	INCOMPLETE – 2	5/26/21	Link to webpage

© 2022 Goodman DC et al. *JAMA Network Open.*

		deliveries, as well as newborns who may require specialized services.”			
Northshore Univ HS Highland Park Hospital (Level II)	Highland Park, IL	“Highland Park is a Level 2 nursery and is able to care for infants 32 weeks and greater. Neonatology is available for consultation when needed for your infant”	ACCURATE	5/26/21	Link to webpage
Northwestern Medicine Huntley Hospital (Level II)	Huntley, IL	“In addition to Onsite’s team of neonatologists, the unit has a specially trained team of care providers dedicated to providing your baby the highest level of specialized care possible.”	INACCURATE – 5	5/26/21	Link to webpage
Northwestern Medicine Kishwaukee Hospital (Level II)	Dekalb, IL	"Our level II labor and delivery services allow us to care for moms and babies who need extended hospitalization or have special needs."	INCOMPLETE – 2	5/26/21	Link to webpage
Northwestern Medicine Valley West Hospital (Level II)	Sandwich, IL	Same description as above.	INCOMPLETE – 2	5/26/21	Link to webpage
Norwegian-American Hospital (Level II)	Chicago, IL	“Our leading Family Birthing Center is fully prepared to assist any pregnancy from “normal” to multiple to high-risk with an expert neonatal team and intensive care unit.”	INACCURATE – 4	5/26/21	Link to webpage
OSF Sacred Heart Medical Center (Level II)	Danville, IL	"If your baby needs more intensive care, we're ready with Level II plus special care nursery services where infants are cared for by specially trained nurses”	INACCURATE – 1	5/26/21	Link to Hospital web site
OSF Saint Anthony Medical Center (Level II)	Rockford, IL	No reference to unit.	INCOMPLETE – 1	5/26/21	Link to Hospital web site
OSF Saint Elizabeth Medical Center (Level II)	Ottawa, IL	"Other features of our Family Birth Center include: Level 2 nursery"	INCOMPLETE – 2	5/26/21	Link to webpage
OSF St Joseph Medical Center (Level II)	Bloomington, IL	“"These specialists provide the highest level of expertise around-the-clock for all of our newborns and will do so throughout your newborn’s stay in our Birthing Center. This added level of expert care allows babies who may have been	INACCURATE – 5	5/26/21	Link to webpage

© 2022 Goodman DC et al. *JAMA Network Open.*

		transferred to OSF Children's Hospital in the past to remain right here close to home in the nursery"			
OSF St Mary Medical Center (Level II)	Galesburg, IL	No reference to unit.	INCOMPLETE – 1	5/26/21	Link to Hospital web site
Palos Community Hospital (Level II)	Palos Heights, IL	No reference to unit.	INCOMPLETE – 1	5/26/21	Link to Hospital web site
Passavant Area Hospital (Level II)	Jacksonville, IL	"Level II Nursery: If for any reason your baby is in the Level II Nursery, requiring more comprehensive care"	INCOMPLETE – 2	5/26/21	Link to webpage
Richland Memorial Hospital (Level II)	Olney, IL	No reference to unit.	INCOMPLETE – 1	5/26/21	Link to Hospital web site
Riverside Medical Center (Level II)	Kanakee, IL	"Our obstetrics and maternity facilities are equipped with a Level II Special care nursery … if your baby is born significantly early or has medical complications, he or she will be transferred to a Level III NICU."	ACCURATE	5/26/21	Link to webpage
Roseland Community Hospital (Level II)	Chicago, IL	No reference to unit.	INCOMPLETE – 1	5/26/21	Link to Hospital web site
Sarah Bush Lincoln Health Center (Level II)	Mattoon, IL	"a Level II Perinatal Unit"	INCOMPLETE – 2	5/26/21	Link to webpage
SSM Health Good Samaritan Hospital (Level II)	Mount Vernon, IL	No reference to unit.	INCOMPLETE – 1	5/26/21	Link to Hospital web site
SSM Health St Mary's Hospital (Level II)	Centralia, IL	No reference to unit.	INCOMPLETE – 1	5/26/21	Link to Hospital web site
St Anthony Hospital (Level II)	Chicago, IL	"Skilled neonatologists provide excellent, attentive care to our newborns in our Level II nursery. Babies needing a higher level of care can be transferred to the Neonatal Intensive Care Unit	ACCURATE	5/26/21	Link to webpage

© 2022 Goodman DC et al. *JAMA Network Open.*

		(NICU) at the University of Chicago Comer Children's”			
St Margaret's Health (Level II)	Spring Valley, IL	No reference to unit.	INCOMPLETE – 1	5/26/21	Link to Hospital web site
Swedish Covenant Hospital (Level II)	Chicago, IL	“In general, care in our ICN is usually limited to newborn infants who are more than 32 weeks gestational age and weigh more than 1,500 grams at birth or who are recovering from serious illness treated in a Level III NICU. Newborns that are seriously ill may be transferred to a facility with a Level III NICU. "	ACCURATE	5/26/21	Link to webpage
Vista Medical Center East (Level II)	Waukegan, IL	No reference to unit.	INCOMPLETE – 1	5/26/21	Link to Hospital web site
West Suburban Medical Center (Level II)	Oak Park, IL	“Neonatologists provide the most advanced care to premature and critically ill infants and are on staff at West Suburban Medical Center's Level II Nursery."	INACCURATE – 5	5/26/21	Link to webpage
Advocate Condell Medical Center (Level II+)	Libertyville, IL	"Level II special care nursery: We can care for babies as low as 30 weeks gestation with high acuity. The nursery is affiliated with the Northshore Perinatal Network and Advocate Children's Hospital, allowing us to provide immediate access to specialized treatment”	ACCURATE	5/26/21	Link to webpage
Advocate Good Shepherd Hospital (Level II+)	Barrington IL	“Our level II extended neonatal capabilities perinatal care facility gives you and your baby access to the highest level of neonatal and pediatric care available in Illinois”	INACCURATE – 5	5/26/21	Link to webpage
Advocate Sherman Hospital (Level II+)	Elgin, IL	“Our Level II Extended (IIE) special care nursery … with the necessary staff, services, equipment and demonstrated record of excellent outcomes in treating high-risk newborns, including premature or low-weight babies.”	ACCURATE	5/26/21	Link to webpage
AMITA Health Elk Grove Village - Alexian Brothers Medical Center (Level II+)	Elk Grove Village, IL	"Sometimes called IIE (for "extended"), Level II+ Perinatal Services offers the same care capabilities as Level II, but with the added support of a special care nursery."	INCOMPLETE – 2	5/26/21	Link to webpage

© 2022 Goodman DC et al. *JAMA Network Open.*

AMITA Resurrection Medical Center (Level II+)	Chicago, IL	Same description as above.	INCOMPLETE – 2	5/26/21	Link to webpage
AMITA Saints Mary and Elizabeth Medical Center - St. Mary of Nazareth Campus (Level II+)	Chicago, IL	Same description as above.	INCOMPLETE – 2	5/26/21	Link to webpage
AMITA St. Joseph's Medical Center (Level II+)	Joliet IL	Same description as above.	INCOMPLETE – 2	5/26/21	Link to webpage
Centegra McHenry Hospital (Level II+)	McHenry IL	"We have partnered with Onsite Neonatal Partners … to providing your baby the highest level of specialized care possible."	INACCURATE – 5	5/26/21	Link to webpage
Elmhurst Memorial Hospital (Level II+)	Elmhurst IL	"Your baby will have access to … extended capabilities in the Level IIe Special Care Nursery at Elmhurst Hospital."	INCOMPLETE – 2	5/26/21	Link to webpage
Franciscan St. James Olympia Fields Hospital (Level II+)	Olympia Fields IL	Does not state level of neonatal unit.	INCOMPLETE – 1	5/26/21	Link to webpage
Ingalls Memorial Hospital (Level II+)	Harvey IL	"Our Level 2 nursery provides specialized newborn care with extended neonatal capabilities. Our NICU staff is extensively trained to care for babies born at 30 weeks and older."	ACCURATE	5/26/21	Link to webpage
Little Company of Mary Hospital (Level II+)	Evergreen Park IL	"Infants who require special attention following birth are cared for in our special care nursery. In addition, neonatal specialists are available 24 hours a day to give these infants the treatment and attention they deserve"	INCOMPLETE – 2	5/26/21	Link to webpage
Memorial Hospital EAST	Shiloh IL	"The Level II E Nursery has everything the Level II Nursery does, but its team also cares for babies	ACCURATE	5/26/21	Link to webpage

© 2022 Goodman DC et al. *JAMA Network Open.*

(Level II+)		who are a little earlier (as early as 30 weeks), a little smaller (as small as 2.75 pounds), and a little more fragile (those on assisted ventilation)."			
Memorial Hospital of Carbondale (Level II+)	Carbondale IL	"Level II Plus Special Care Nursery, which means we can take care of babies as early as 28 weeks gestation"	ACCURATE	5/26/21	Link to webpage
Mercy Hospital and Medical Center (Level II+)	Chicago IL	"If a new baby requires special care, Mercy offers a Specialty Care Unit, where skilled neonatologists deliver care for premature and special needs babies."	INCOMPLETE – 2	5/26/21	Link to webpage
Northwestern Medicine Delnor Hospital (Level II+)	Geneva IL	"Northwestern Medicine Delnor Hospital offers a Level II-E Special Care Nursery"	INCOMPLETE – 2	5/26/21	Link to webpage
Northwestern Medicine Lake Forest Hospital (Level II+)	Lake Forest IL	No reference to unit.	INCOMPLETE – 1	5/26/21	Link to Hospital Web site
Presence Covenant Medical Center (Level II+)	Urbana IL	"We're ready with Level II plus Special Care Nursery services. Our team of neonatologists is on-site around the clock ready to respond with specialized care for babies born at 30 weeks and above."	ACCURATE	5/26/21	Link to webpage
Silver Cross Hospital (Level II+)	Lemont IL	No reference to unit.	INCOMPLETE – 1	5/26/21	Link to webpage
UnityPoint Health - Methodist (Level II+)	Peoria IL	"If your baby requires more advanced care, our certified Level II Nursery with Extended Neonatal capabilities cares for babies born at 32 weeks' gestation or later."	ACCURATE	5/26/21	Link to webpage
UnityPoint Health - Trinity (Level II+)	Moline IL	"Level II NSCU provides expert medical care for babies who are sick or who are born too early (before 37 weeks of pregnancy). We offer private bays for families as well as several private rooms to accommodate twins or triplets."	ACCURATE	5/26/21	Link to webpage
Adena Health System	Chillicothe OH	"Certified physicians and nurses provide around-the-clock specialized services in our Level II Special Care Unit. also offer central monitoring	ACCURATE	5/25/21	Link to webpage

© 2022 Goodman DC et al. *JAMA Network Open.*

		capabilities, IV therapy, photo therapy, a respiratory simulator, and more."			
Atrium Medical Center	Middletown OH	“Premier Health Special Care Nursery is a Level II nursery. This means they’re equipped to address and manage complications that regular nurseries can’t. Conditions managed include: Low birth weight/premature birth; Genetic conditions; Respiratory difficulties”	ACCURATE	5/25/21	Link to webpage
Bethesda North Hospital	Montgomery OH	"At the Level II Harold and Margret Thomas Special Care Nursery, you can rest assured that your baby is receiving the most advanced, evidence-based medical and nursing care available."	INACCURATE – 5	5/25/21	Link to webpage
Blanchard Valley Hospital	Findlay OH	“Blanchard Valley Hospital is designated as a Level 2 nursery, meaning we can care for most babies born at 32 weeks gestation or later – allowing most infants born at Blanchard Valley Hospital to stay close to home.”	ACCURATE	5/25/21	Link to webpage
Christ Hospital Medical Center – Liberty	Liberty Township OH	"Level II Special Care Nursery "	INCOMPLETE – 2	5/25/21	Link to webpage
Dublin Methodist Hospital	Dublin OH	"Nationwide Children's Hospital operates our Level II Neonatal Special Care Nursery.”	INCOMPLETE – 2	5/25/21	Link to webpage
Fort Hamilton Hospital	Hamilton OH	Our Newly Renovated Special Care Nursery offers: Neonatology services available 24/7; Neonatal respiratory team available; Special care nurses; State-of-the art equipment”	ACCURATE	5/25/21	Link to webpage
Genesis Hospital	Zanesville OH	"The Level II Special Care Nursery at Genesis Hospital cares for babies born as early as 32 weeks gestation. In addition, the Special Care Nursery cares for full-term babies with special needs.	ACCURATE	5/25/21	Link to webpage
Licking Memorial Hospital	Newark OH	"In special circumstances, our Special Care Nursery provides a safe, secure environment for … ill newborns who require intermediate care … born as early as 32 weeks gestation, or infants who require medical assistance."	ACCURATE	5/25/21	Link to webpage

© 2022 Goodman DC et al. *JAMA Network Open.*

Lima Memorial Hospital	Lima OH	"Lima Memorial Hospital's Level II Neonatal Nursery treats babies 32 weeks gestation and older. you have. Those in need of the most critical level of care receive exactly what they require, thanks to our partnership with Toledo Children's Hospital and their Level III Neonatal Nursery"	ACCURATE	5/25/21	Link to webpage
Mercy Health – Fairfield Hospital	Fairfield OH	This hospital network does not have individual web sites for each hospital. It describes the services the hospitals offer generally and does not specify which hospital offers which services<br><br>"Most babies are born healthy and full of wonder. However, sometimes the need arises for more advanced care. For babies born prematurely or those having special health needs, we have a Neonatal Intensive Care Unit (NICU) or special care nursery available."	INACCURATE – 4	5/25/21	Link to webpage
Mercy Health – Lorain Hospital	Lorain OH	Same description as above	INACCURATE – 4	5/25/21	Link to webpage
Mercy Health – West Hospital	Cincinnati OH	Same description as above	INACCURATE – 4	5/25/21	Link to webpage
Mercy Health Hospital Anderson	Anderson OH	Same description as above	INACCURATE – 4	5/25/21	Link to webpage
Mercy Health St. Rita's Medical Center	Lima OH	Same description as above	INACCURATE – 4	5/25/21	Link to webpage
Mercy Medical Center	Canton OH	"Mercy Maternity Services is a Level II facility and can handle all mother-baby emergencies."	INACCURATE – 5	5/25/21	Link to webpage
Miami Valley Hospital South	Centerville OH	"Premier Health Special Care Nursery is a Level II nursery. Conditions Level II nurseries manage include: Low birth weight/premature birth; Genetic conditions; Respiratory difficulties; Infections; Drug dependence"	ACCURATE	5/25/21	Link to webpage
Ohio Health Marion General Hospital	Marion OH	"Features a Level II nursery with board certified neonatologists and staff who are trained in treatment programs for select high-risk mothers and newborns. Along with our board certified obstetricians, pediatricians, anesthesiologists, highly-skilled nurses and state-of-the-art	ACCURATE	5/25/21	Link to webpage

© 2022 Goodman DC et al. *JAMA Network Open.*

		technology, our maternity center provides exceptional obstetrical care for you and your baby."			
Ohio Health Mansfield Hospital	Mansfield OH	“Level II special care nursery. Some babies need a little extra care in their first few days of life. And at Mansfield Hospital, they can receive the specialized attention they need to recover and head home.”	INCOMPLETE – 2	5/25/21	Link to webpage
Promedica Bay Park Hospital;	Oregon OH	No reference to unit.	INCOMPLETE – 1	5/25/21	Link to webpage
Soin Medical Center	Beavercreek OH	"Level IIB Special Care Nursery to care for premature babies after 32 weeks or infants that need special care"	INACCURATE – 1	5/25/21	Link to webpage
Southview Medical Center	Montgomery OH	"Level IIB Special Care Nursery to care for premature babies after 32 weeks or infants that need special care"	INACCURATE – 1	5/25/21	Link to webpage
Springfield Regional Medical Center	Springfield OH	This hospital network does not have individual web sites for each hospital. It describes the services the hospitals offer generally and does not specify which hospital offers which services<br><br>“Most babies are born healthy and full of wonder. However, sometimes the need arises for more advanced care. For babies born prematurely or those having special health needs, we have a Neonatal Intensive Care Unit (NICU) or special care nursery available.”	INACCURATE – 4	5/25/21	Link to webpage
St. Joseph Warren Hospital	Warren OH	Same description as above.	INACCURATE – 4	5/25/21	Link to webpage
The Christ Hospital	Cincinatti OH	Expanded Level II Special Care Nursery.<br>Level II Special care nursery for newborns who need more attention after birth	INCOMPLETE – 2	5/25/21	Link to webpage
Upper Valley Medical Center	Troy OH	“Premier Health Special Care Nursery is a Level II nursery. Conditions Level II nurseries manage include: Low birth weight/premature birth; Genetic conditions; Respiratory difficulties; Infections; Drug dependence"	ACCURATE	5/25/21	Link to webpage

© 2022 Goodman DC et al. *JAMA Network Open.*

West Chester Hospital	West Chester Township OH	“West Chester Hospital provides a Level IIB Special Care Nursery for babies who may require additional medical attention.”	INACCURATE – 1	5/25/21	Link to webpage
Wooster Community Hospital	Wooster OH	“Our Level II Special Care Nursery is able to care for babies who: Were born up to 8 weeks premature (at or after 32 weeks’ gestation); Weigh at least 3 pounds, 5 ounces; Are physically immature, such as those who have breathing problems or who are unable to properly feed; Have blood sugar instability”	ACCURATE	5/25/21	Link to webpage
The Chambersburg Hospital	Chambersburg PA	"our NICU Level II at WellSpan Chambersburg Hospital allows babies born as early as 32 weeks gestation or babies with unexpected medical complications to be treated close to home."	INCOMPLETE – 2	5/25/21	Link to webpage
Delaware County Memorial Hospital	Drexel Hill PA	"24/7 NICUs: Both of our maternity centers have state-certified neonatal intensive care units (NICUs) that are fully equipped to care for virtually all high-risk infants around the clock."	INACCURATE – 5	5/25/21	Link to webpage
Doylestown Hospital	Doylestown PA	"If your baby requires a higher level of care due to a premature birth or having a medical complication that involves keen observation and treatment, our Level II Intensive Care Nursery (ICN) has a reputation in the community of providing exceptional care for babies and parents.”	INACCURATE – 4	5/25/21	Link to webpage
Excela Health Westmoreland Hospital	Greensburg PA	“Special Care (Level II) Nursery – for infants born prematurely, with very low birth weight, or other difficulties, we provide ’round-the-clock care by certified neonatal nurse practitioners and neonatologists. And we offer telemedicine consultation through Children’s Hospital of Pittsburgh.”	ACCURATE	5/25/21	Link to webpage
Forbes Hospital	Monroeville PA	“If your baby is born prematurely or has a serious health problem, Forbes and Jefferson hospitals have Level 2 NICUs … Our NICUs serve as regional referral centers, meaning we provide specialized care for infants “	INACCURATE – 4	5/25/21	Link to webpage
Grand View Health	Sellersville PA	“our six-bed Level II NICU is equipped to care for babies born as early as 32 weeks gestation or	ACCURATE	5/25/21	Link to webpage

© 2022 Goodman DC et al. *JAMA Network Open.*

		babies with unexpected medical complications, close to home.”			
Heritage Valley Beaver	Beaver PA	"Heritage Valley Beaver also has a Level II nursery for infants requiring special care"	INCOMPLETE – 2	5/25/21	Link to webpage
Jefferson Hospital	Jefferson PA	“If your baby is born prematurely or has a serious health problem, Forbes and Jefferson hospitals have Level 2 NICUs … Our NICUs serve as regional referral centers, meaning we provide specialized care for infants “	INACCURATE – 4	5/25/21	Link to webpage
Mount Nittany Medical Center	State College PA	No reference to unit.	INCOMPLETE – 1	5/25/21	Link to webpage
Paoli Hospital	Paoli PA	“When you come to the Neonatal Intensive Care Unit (NICU), you can be confident that you and your newborn are in the hands of experts. Each year our four NICU’s care for hundreds of babies who are premature, or low birth rate, have medical problems such as, immature lungs, low blood sugar, respiratory distress, suspected infection or a multitude of other issues.”	INACCURATE – 4	5/25/21	Link to webpage
Penn State Health St. Joseph	Reading PA	No reference to unit.	INCOMPLETE – 1	5/25/21	Link to webpage
Phoenixville Hospital	Phoenixville PA	“Our Level II nursery offers a little extra help for premature newborns with health problems that don’t need advanced care. We also provide care for infants after they leave the NICU and babies who need breathing equipment.”	ACCURATE	5/25/21	Link to webpage
Riddle Memorial Hospital	Media PA	“When you come to the Neonatal Intensive Care Unit (NICU), you can be confident that you and your newborn are in the hands of experts. Each year our four NICU’s care for hundreds of babies who are premature, or low birth rate, have medical problems such as, immature lungs, low blood sugar, respiratory distress, suspected infection or a multitude of other issues.”	INACCURATE – 4	5/25/21	Link to webpage
Robert Packer Hospital	Sayre PA	No reference to unit.	INCOMPLETE – 1	5/25/21	Link to webpage
Sharon Regional Health System	Sharon PA	"6-bed advanced care Level II Nursery, if your baby requires a higher level of care"	INCOMPLETE – 2	5/25/21	Link to webpage

© 2022 Goodman DC et al. *JAMA Network Open.*

St. Clair Hospital	Pittsburgh PA	"If a baby is born premature or ill, a special environment is needed for individualized care and close monitoring … Our Level II Special Care Nursery is specifically designed and equipped to accommodate a baby's needs. Infants in this unit require special care."	ACCURATE	5/25/21	Link to webpage
St. Mary Medical Center	Langhorne PA	"Our state-of-the-art NICU is staffed by board-certified neonatologists (physicians with special training in high-risk newborn care) from Children's Hospital of Philadelphia, and our registered nurses are fully trained in neonatal medicine."	INACCURATE – 4	5/25/21	Link to webpage
UPMC Altoona	Altoona PA	"Continuing Care Nursery. The nursery, found on the same floor as the birthing rooms near mom, is for newborns who need a little extra care. If needed, our pediatricians will refer your baby to a specialist."	INCOMPLETE – 2	5/25/21	Link to webpage
UMPC Carlisle	Carlisle PA	No reference to unit.	INCOMPLETE – 1	5/25/21	Link to webpage
UPMC Horizon	Greenville PA	"Level II nursery"	INCOMPLETE – 2	5/25/21	Link to webpage
UPMC Memorial	York PA	PMC Memorial has a Level II NICU where specialty care can be given to babies born at 32 weeks gestation and above. We deliver round-the-clock treatment for a number of conditions, including: Breathing problems; Premature birth; Conditions affecting the brain; Heart conditions; Stomach and digestive disorders…"	ACCURATE	5/25/21	Link to webpage
UPMC Northwest	Seneca PA	"Three-bed Level II nursery"	INCOMPLETE – 2	5/25/21	Link to webpage
UPMC Williamsport	Williamsport PA	"Our specially trained Level II medical staff includes board-certified pediatricians and nurses. They care for premature infants born at 32 weeks of gestation or older (at least 1500 gms) and full-term babies with medical conditions, such as the need for special respiratory care"	ACCURATE	5/25/21	Link to webpage
The Washington Hospital	Washington PA	"a nursery with a Level 2 special care area for infants with special medical needs."	INCOMPLETE – 2	5/25/21	Link to webpage
Wilkes-Barre General Hospital	Wilkes – Barre PA	No reference to unit.	INCOMPLETE – 1	5/25/21	Link to webpage

© 2022 Goodman DC et al. *JAMA Network Open.*

Atrium Health Cleveland	Shelby NC	No reference to unit.	INCOMPLETE – 1	5/26/21	Link to webpage
Atrium Health Lincoln	Lincolnton NC	"In addition to our newborn nursery, we also have a level 2 nursery for babies requiring special care."	INCOMPLETE – 2	5/26/21	Link to webpage
Betsy Johnson Hospital	Dunn NC	No reference to unit.	INCOMPLETE – 1	5/26/21	Link to webpage
Carolinas Medical Center	Charlotte NC	"the largest neonatal intensive care nursery in the Carolinas, as well as the only ECMO (heart/lung respiratory assistance) system in the region. Carolinas Medical Center provides 24-hour neonatologist, perinatologist and anesthesiologist care as well as around the clock availability of every kind of specialist imaginable."	INACCURATE – 4	5/26/21	Link to webpage
Carteret General Hospital	Morehead City NC	"Our intermediate nursery are available for babies who need a little extra help following birth. Services include: cardiac monitoring, respiratory and oxygen therapy, and nutritional support to provide for your infant's continuing growth and development."	ACCURATE	5/26/21	Link to webpage
Central Carolina Hospital	Sanford NC	"The Level Two nursery provides care to well neonates and also those who require supportive care. Those infants who require intubation and ventilation are stabilized with the proper equipment and then transferred to a tertiary care facility. The nursery is equipped with Isolettes to care for stable premature infants who require time to grow"	ACCURATE	5/26/21	Link to webpage
Duke Regional Hospital	Durham NC	"Care for about 400 infants every year who are born prematurely as well as babies born with common neonatal conditions such as breathing problems or suspected infections. If your baby is delivered at less than 32 weeks gestation or requires surgery or a higher level of care, he or she will immediately be transported"	ACCURATE	5/26/21	Link to webpage
Duke University Hospital	Durham NC	"Infants with complex problems are cared for in Duke University Hospital's Level IV Neonatal Intensive Care Unit (NICU). The Level IV designation indicates we provide the highest level of care for critically ill infants."	INACCURATE – 3	5/26/21	Link to webpage

© 2022 Goodman DC et al. *JAMA Network Open.*

Granville Health System	Oxford NC	No reference to unit.	INCOMPLETE – 1	5/26/21	Link to webpage
Johnston Health	Smithfield NC	"If your newborn needs a little extra time in the hospital to grow strong and healthy, you'll appreciate the Level II nursery at Johnston Health in Smithfield. This special care nursery tends to premature or moderately sick babies born at least 32 weeks gestation."	ACCURATE	5/26/21	Link to webpage
Maria Parham Health	Henderson NC	No reference to unit.	INCOMPLETE – 1	5/26/21	Link to webpage
Novant Health Rowan Medical Center	Salisbury NC	Page describes NICUs in the NovantHealth system generally and does not specify whether Rowan has a NICU or special care nursery	INCOMPLETE – 1	5/26/21	Link to webpage
Scotland Memorial Hospital	Laurinburg NC	"Within the Special Care Nursery there are five bassinets in an enclosed area where the specially trained staff care for infants with additional needs after delivery. Equipped with the following: Three radiant warmer beds; Three infant incubators; Five cardiac/apnea monitors; Three pulse oximeters; Emergency resuscitative drugs for neonates"	ACCURATE	5/26/21	Link to webpage
Southeastern Health	Lumberton NC	Has a unit but does not state the level or describe its services. There is a NICU virtual tour on the maternity center web site, but it does not provide any description of services.	INACCURATE – 4	5/26/21	Link to webpage
The Outer Banks Hospital	Nags Head NC	No reference to unit.	INCOMPLETE – 1	5/26/21	Link to webpage
Vidant Medical Center	Greenville NC	No reference to unit.	INCOMPLETE – 1	5/26/21	Link to webpage
Watauga Medical Center, Inc.	Boone NC	"Level 2 Newborn Nursery"	INCOMPLETE – 2	5/26/21	Link to webpage
Wayne UNC Health Care	Goldsboro NC	"Rely on our Level II NICU to provide newborn care for babies born at 32 weeks gestational age up to full-term babies that need special attention and intervention. Care and treatment at Wayne UNC includes: Close medical monitoring; Oxygen	ACCURATE	5/26/21	Link to webpage

		support; Intravenous (IV) antibiotics for infection; Phototherapy; Special feeding"			
Wilson Medical Center	Wilson NC	No reference to unit.	INCOMPLETE – 1	5/26/21	Link to webpage
CarePoint Health-Hoboken University Medical Center	Hoboken NJ	"special care nursery can care for babies born after 32 weeks gestational age who: Can't stay warm on their own and need to be placed under a warmer; Aren't strong enough to eat well and need support and assistance; Have mild health problems related to prematurity, such as jaundice or apnea of prematurity; Are preparing to be transported to a Level III NICU; Are recovering after having spent time in a Level III NICU"	ACCURATE	5/25/21	Link to webpage
Centra State Medical Center	Freehold NJ	"Special Care Nursery at CentraState has a dedicated staff of neonatologists and highly skilled nurses available 24/7. They provide individualized care for premature babies born after 32 weeks of pregnancy who require close observation or other special attention."	ACCURATE	5/25/21	Link to webpage
Chilton Medical Center	Pompton Plains NJ	"Level II Special Care Nursery, an additional setting for high quality neonatal care within Atlantic Health System. It is always staffed by a team of experienced neonatologists, advanced neonatal practitioners and pediatric hospitalists. The SCN provides care to infants born at a gestational age of greater than 31 weeks who require critical care, including infants with infections and respiratory problems."	ACCURATE	5/25/21	Link to webpage
Clara Maass Medical Center	Belleville NJ	"In addition to our Newborn Nursery, Clara Maass Medical Center maintains a Level II Special Care Nursery for premature newborns born at 32 weeks or greater, and newborns in need of specialized medical treatment. With a neonatologist that is available 24-hours a day, and a highly skilled team of neonatal nurses"	ACCURATE	5/25/21	Link to webpage
Community Medical Center	Toms River NJ	"A newborn nursery and a dedicated Level 2 special care unit for infants requiring special attention is also available, and neonatologists are onsite 24/7."	INCOMPLETE – 2	5/25/21	Link to webpage

© 2022 Goodman DC et al. *JAMA Network Open.*

HackensackMeridian Health, Mountainside Medical Center	Montclair NJ	"The mission of our Neonatology Department is to provide superior care to infants in the well newborn nursery and specialized care for ill or premature infants in our state-of-the-art special care nursery. "	INCOMPLETE – 2	5/25/21	Link to webpage
Hudson Regional Hospital	Seacaucus NJ	"All OB/GYN staff go out of their way to make your hospital stay and birthing experience as comfortable as possible. Our newborn nursery includes four designated intermediate-level (Level II) nursery cribs for intermediate-level care to pre-term and normal newborns requiring more specialized care."	INCOMPLETE – 2	5/25/21	Link to webpage
Hunterdon Medical Center	Flemington NJ	"The Special Care Nursery provides expert care for newborns who are born up to eight weeks premature or with a medical problem, such as an infection or respiratory condition … Any baby born prior to 32 weeks can be readied for transport to the nearest NICU"	ACCURATE	5/25/21	Link to webpage
JFK University Medical Center	Edison NJ	"For babies requiring extra care at birth, we offer a state-of-the-art, Level 2 Special Care Nursery."	INCOMPLETE – 2	5/25/21	Link to webpage
Newton Medical Center	Newton NJ	"Newton Medical Center also offers specialized neonatal care in a Level II Intermediate Care Nursery staffed by in-house pediatric hospitalists, available around the clock to coordinate neonatal care and attend deliveries. The SCN provides care to infants born at a gestational age of greater than 31 weeks who require critical care, including infants with infections and respiratory problems."	ACCURATE	5/25/21	Link to webpage
Ocean Medical Center	Brick NJ	"Pediatric services also extend to the maternity unit. Ocean Medical Center offers Level II neonatology for infants who need more intensive observation after birth."	INCOMPLETE – 2	5/25/21	Link to webpage
Palisades Medical Center	North Bergen NJ	"For parents-to-be, nothing is more important than the health of their baby. It's comforting to know that our Maternity Center features a certified Level 2 Neonatal Nursery to care for babies should health problems arise. Level 2 Nursery is always staffed by one or more board certified neonatologists, resident physicians, nurses, nurse	ACCURATE	5/25/21	Link to webpage

© 2022 Goodman DC et al. *JAMA Network Open.*

		practitioners, pharmacists, physician assistants, and respiratory therapists.”			
Pascack Valley Medical Center, Hackensack Meridian Health	Westwood NJ	"Our state-of-the-art center features 6 Labor and Delivery rooms; 18 private post-partum rooms, each with private, hotel-like bath; 2 C-section suites; and a Level II NICU for babies needing extra support. "	INCOMPLETE – 2	5/25/21	Link to webpage
Raritan Bay Medical Center Perth Amboy Division	Perth Amboy NJ	"Enveloped within a warm, spacious, nurturing environment, the unit features birthing suites that allow mothers to labor, deliver and recover in one place, as well as surgical suites and a level II neonatal special care nursery."	INCOMPLETE – 2	5/25/21	Link to webpage
Riverview Medical Center	Red Bank NJ	“For babies requiring extra care at birth, Riverview offers a state-of-the-art, Level II neo-natal intensive care unit (NICU), also known as a Special Care Nursery. The NICU treats moderately premature babies and full-term babies that require close monitoring of health issues.”	ACCURATE	5/25/21	Link to webpage
Robert Wood Johnson Hospital Somerset	Somerville NJ	“RWJ Somerset is home to a Level II Intermediate Care Nursery in addition to the well-baby nursery. There is an RWJ neonatologist present 24/7 to provide specialized medical care for infants born at 32 weeks or more. The nursery uses the latest technologies and monitoring devices to care for your baby.”	ACCURATE	5/25/21	Link to webpage
Saint Claire's Hospital/Denville Campus	Denville NJ	“Level II Intermediate Care Nursery houses eight bassinets and is equipped with state-of-the-art equipment, advanced monitoring and charting systems, private rooms for moms to breastfeed or bond, and a separate family waiting area … for the newborn nursery, NICU, labor and delivery, and any admission of a baby 28 days and younger”	ACCURATE	5/25/21	Link to webpage
Shore Medical Center	Sommers Point NJ	“Shore’s Special Care Nursery, supported by Onsite neonatologists, is equipped to handle newborn deliveries as early as 32 weeks and provides highly specialized care. Our Special Care Nursery team uses high-tech monitoring devices for sick or special-care newborns,	ACCURATE	5/25/21	Link to webpage

© 2022 Goodman DC et al. *JAMA Network Open.*

		allowing these babies to remain with the family rather than requiring transfer to another hospital."			
St Joseph's Wayne Medical Center	Wayne NJ	"High-risk infants are cared for by Neonatal Associates of St. Joseph's University Medical Center. These Board-certified physicians have a long history of caring for the tiniest of newborns."	INACCURATE – 5	5/25/21	Link to webpage
St. Mary's General Hospital	Passaic NJ	"The fully renovated Maternal-Child Health Center at St. Mary's General Hospital features a Level II Nursery specializing in the delivery and care of high-risk infants born as early as 32 weeks."	ACCURATE	5/25/21	Link to webpage
Trinitas Regional Medical Center	Elizabeth NJ	"Our Obstetrical Services consist of Labor & Delivery, a Level II Intermediate Care Nursery, and a Mother/Baby Unit. Our Level II Nursery offers sophisticated technology and treatment for ill and high-risk babies who require concentrated care and attention."	INCOMPLETE – 2	5/25/21	Link to webpage
Virtua Memorial Hospital	Mount Holly NJ	"our NICU and SCN are staffed by teams of board-certified CHOP at Virtua neonatologists (pediatricians with advanced training in newborn intensive care), neonatal nurse practitioners and neonatal nurses. Our team offers advanced expertise in the complex care of the tiniest and sickest babies, and they do so with the greatest compassion and skill."	INACCURATE – 5	5/25/21	Link to webpage
Virtua Our Lady of Lourdes Hospital	Camden NJ	Same description as above.	INACCURATE – 5	5/25/21	Link to webpage
Augusta Health	Fisherville VA	No reference to unit.	INCOMPLETE – 1	5/25/21	Link to webpage
Bon Secours Southside Medical Center	Petersburg VA	No reference to unit.	INCOMPLETE – 1	5/25/21	Link to webpage
Carlion New River Valley Medical Center	Christiansburg VA	No reference to unit.	INCOMPLETE – 1	5/25/21	Link to webpage
Fauquier Hospital	Warrenton VA	"The neonatal ICN -- designed to provide care for babies who are born at Fauquier Hospital but are born prematurely or are too sick to go home right away -- means that parents of these babies can	ACCURATE	5/25/21	Link to webpage

© 2022 Goodman DC et al. *JAMA Network Open.*

		be with their newborns every day without having to travel to Northern Virginia or Charlottesville. Some infants will still need the care that can only be provided in a Neonatal Intensive Care Unit – a higher level of care -- but many babies will be able to remain in Fauquier, near their loved ones."			
Johnston Memorial Hospital	Abingdon VA	No reference to unit.	INCOMPLETE – 1	5/25/21	Link to webpage
Lewis Gale Hospital Montgomery	Blacksburg VA	“home to a Level II Neonatal Intensive Care Unit (NICU), also called a special care nursery. Our hospital in Blacksburg, Virginia, is proud to offer specialized newborn care for infants with minor neonatal concerns and babies born between 32 and 35 weeks of gestation. Our NICU is equipped to care for newborns experiencing mild health problems such as jaundice or smaller-than-average infants. In the event advanced care is needed, we provide transport to one of our partner facilities."	ACCURATE	5/25/21	Link to webpage
Norton Community Hospital	Norton VA	No reference to unit.	INCOMPLETE – 1	5/25/21	Link to webpage
Novant Health UVA – Haymarket Medical Center	Manassas VA	"If your baby is born prematurely or with an illness or condition that requires special care… We have teams of board-certified neonatologists, nurse practitioners, registered nurses, respiratory therapists, social workers, pharmacists, nutritionists and lactation consultants on staff in our intermediate care nursery (ICN) … Babies who need a higher level of care will be … transportation to a higher-level facility."	ACCURATE	5/25/21	Link to webpage
Riverside Shore Memorial Hospital	Onancock VA	No reference to unit.	INCOMPLETE – 1	5/25/21	Link to webpage
Sentara Careplex Hospital	Hampton VA	No reference to unit.	INCOMPLETE – 1	5/25/21	Link to webpage
Sentara Leigh Hospital	Norfolk VA	"Services include labor and delivery care, postpartum care, newborn and special care	INCOMPLETE – 2	5/25/21	Link to webpage

© 2022 Goodman DC et al. *JAMA Network Open.*

		nursery, breastfeeding, gynecology and women's health classes"			
Sentara Martha Jefferson Hospital	Charlottesville VA	"We offer a safe and secure atmosphere for you to deliver your baby. Our maternity hospital includes a Special Care Nursery.	INCOMPLETE – 2	5/25/21	Link to webpage
Sentara Obici Hospital	Suffolk VA	No reference to unit.	INCOMPLETE – 1	5/25/21	Link to webpage
Sentara RMH Medical Center	Harrisonburg VA	No reference to unit.	INCOMPLETE – 1	5/25/21	Link to webpage
Sentara Williamsburg Regional Medical Center	Williamsburg VA	"an intermediate care nursery"	INCOMPLETE – 2	5/25/21	Link to webpage
Stafford Hospital	Stafford VA	"Stafford Hospital has a 6-bed, Level II Nursery, also referred to as an Intermediate Level Nursery (ILN). It is equipped with the latest neonatal technology to provide care for infants who are delivered at 34 weeks or later and weigh at least 3.3 pounds, or 1500 grams. The ILN team can provide care for newborns that need extra medical attention but aren't sick enough to need critical care"	ACCURATE	5/25/21	Link to webpage
Stone Springs Hospital Center	Dulles VA	"Level II Neonatal Intensive Care Unit, where we care for babies born prematurely or with other health needs"	INACCURATE – 4	5/25/21	Link to webpage
Twin County Regional Hospital	Galax VA	No reference to unit.	INCOMPLETE – 1	5/25/21	Link to webpage

© 2022 Goodman DC et al. *JAMA Network Open.*

eTable 2. Level II Advanced Care Nurseries in the 10 States With Highest Live Births and State Designation Regulations

				State regulations					
	**Live births (2019)**	**Total Advanced care nurseries**	**Level II units**	**Term for unit**	**Source of Level II List**	**GA or weight neonate limits**	**Acuity limits**	**Specifies limits on duration or type of care**	**Clinician requirements**
California	446,479	125	14 (11%)	Neonatal Intensive Care Unit (Intermediate)	Easily available online	Refers to AAP guidelines	Y	Y	Neonatologist or pediatrician
Texas	377,599	134	54 (43%)	Special care nursery	Easily available online	Y	Y	Y	Not specified
New York	221,539	76	25 (33%)	Level II Perinatal Center	Easily available online	Y	Y	Y	Neonatologist within 20 min.
Florida	220,002	73	35 (49%)	Level II Neonatal Intensive Care Unit	Required submission of a formal request	Y	N	Y "ill" neonates, as opposed to "severely ill" for Level III	Neonatologist
Illinois	140,128	77	50 (71.9%)	Perinatal Level II or II+	Difficult to find online	Y	Y	Y	Neonatologist (level II+)
Ohio	134,461	53	27 (52%)	Level II neonatal care service	Provided with call or email	N	Y	Y	Neonatologist
Pennsylvania	134,230	57	21 (41%)	Specialty-level facility (level II) or Special Care Nursery	Required submission of a formal request	Y	Y	Refers to AAP guidelines	Not specified
North Carolina	118,725	46	11 (34.5%)	Level II Neonatal Service	Provided with call or email	N	Y	N	Physician with training
New Jersey	99,585	44	23 (53%)	Intermediate Care Nursery	Difficult to find online	Y	N	Y	Certified neonatal or

© 2022 Goodman DC et al. *JAMA Network Open.*

									pediatric nurse practitioner
Virginia	97,429	32	8 (43%)	Intermediate Level Newborn Service	Required submission of a formal request	N	Y	N	Pediatrician with training
Total	1,990,177	717	268 (37.3%)						

© 2022 Goodman DC et al. *JAMA Network Open.*

eTable 3. Inaccurate and Incomplete Web Descriptions of Level II Advanced Care Nurseries for 10 Large States, 2021

	CA	TX	NY	FL	IL Level II (2)	IL Level II+	OH	PA	NC	NJ	VA	Total
**Number of Level II units (1)**	14	54	25	35	30	20	27	21	11	23	8	268
**Inaccurate or Incomplete description, no. (%)**	**9 (64.3)**	**40 (74.1)**	**15 (60.0)**	**26 (74.3)**	**18 (60.0)**	**12 (60.0)**	**16 (59.3)**	**15 (71.4)**	**5 (45.5)**	**11 (47.8)**	**4 (50)**	**171 (63.8)**
**Inaccurate description, no. (%)**	**7 (50.00)**	**30 (55.6)**	**13 (52.0)**	**21 (60.0)**	**7 (23.3)**	**2 (10.0)**	**12 (44.4)**	**7 (33.3)**	**2 (18.2)**	**3 (13.0)**	**1 (12.5)**	**105 (39.2)**
Identified as Level III unit	0	2 (3.7)	0	1 (2.9)	2 (6.7)	0	3 (11.1)	0	0	0	0	8 (3.0)
Identified as Level II and Level III unit	0	3 (5.6)	0	0 (0.0)	1 (3.3)	0	0	0	0	0	0	4 (1.5)
Uses term Neonatal Intensive Care Unit or NICU w/o indicating limits in 1) the care available or 2) the degree of prematurity/acuity/complexity of newborns	4 (28.6)	21 (38.9)	12 (48.0)	14 (40.0)	1 (3.3)	0	7 (25.9)	6 (28.6)	2 (18.2)	0	1 (12.5)	68 (25.4)
Description indicating that unit provides the highest or most advanced level of care, without qualifiers	3 (21.4)	4 (7.4)	1 (4.0)	6 (17.1)	3 (10.0)	2 (10.0)	2 (7.4)	1 (4.8)	0	3 (13.0)	0	25 (9.3)
**Incomplete description, no. (%)**	**2 (14.3)**	**10 (18.5)**	**2 (8.0)**	**5 (14.3)**	**11 (36.7)**	**10 (50.0)**	**4 (14.8)**	**8 (38.1)**	**3 (27.3)**	**8 (34.8)**	**3 (37.5)**	**66 (24.6)**
No mention of unit	0	1 (1.9)	0 (0)	2 (5.7)	0 (0.0)	2 (10.0)	0	1 (4.8)	1 (9.1)	0	0	7 (2.6)
Identified as Level II unit but without description	2 (14.3)	9 (16.7)	2 (8.0)	3 (8.6)	11 (36.7)	8 (40.0)	4 (14.8)	7 (33.3)	2 (18.2)	8 (34.8)	3 (37.5)	59 (22.0)
Notes: (1) State terms for Level II units varies.												
(2) Illionois has Level II and Level II+ units.												

© 2022 Goodman DC et al. *JAMA Network Open.*
